# Nuclear hormone receptor NHR-49 is an essential regulator of stress resilience and healthy aging in *Caenorhabditis elegans*

**DOI:** 10.3389/fphys.2023.1241591

**Published:** 2023-08-14

**Authors:** Kelsie R. S. Doering, Glafira Ermakova, Stefan Taubert

**Affiliations:** ^1^ Centre for Molecular Medicine and Therapeutics, The University of British Columbia, Vancouver, BC, Canada; ^2^ Edwin S. H. Leong Centre for Healthy Aging, The University of British Columbia, Vancouver, BC, Canada; ^3^ British Columbia Children’s Hospital Research Institute, Vancouver, BC, Canada; ^4^ Department of Medical Genetics, The University of British Columbia, Vancouver, BC, Canada

**Keywords:** *nhr-49*, HNF4, PPAR, longevity, GLP-1, fatty acid desaturation, stress response, fatty acid β oxidation

## Abstract

The genome of *Caenorhabditis elegans* encodes 284 nuclear hormone receptor, which perform diverse functions in development and physiology. One of the best characterized of these is NHR-49, related in sequence and function to mammalian hepatocyte nuclear factor 4α and peroxisome proliferator-activated receptor *α*. Initially identified as regulator of lipid metabolism, including fatty acid catabolism and desaturation, additional important roles for NHR-49 have since emerged. It is an essential contributor to longevity in several genetic and environmental contexts, and also plays vital roles in the resistance to several stresses and innate immune response to infection with various bacterial pathogens. Here, we review how NHR-49 is integrated into pertinent signaling circuits and how it achieves its diverse functions. We also highlight areas for future investigation including identification of regulatory inputs that drive NHR-49 activity and identification of tissue-specific gene regulatory outputs. We anticipate that future work on this protein will provide information that could be useful for developing strategies to age-associated declines in health and age-related human diseases.

## 1 *Caenorhabditis elegans* nuclear hormone receptors control metabolism, stress responses, and aging

Nuclear hormone receptors (NHRs) are a family of metazoan transcription factors whose activity can be altered by ligands, including steroid hormones, fatty acid like molecules, and other compounds (Sever and Glass, 2013; Weikum et al., 2018; Frigo et al., 2021). The roles of NHRs include the regulation of animal development, growth, proliferation, physiology, metabolism, stress response, aging, and others. Their diverse and context-specific impact on gene regulation, altered activity in many pathological states, and accessibility to pharmacological modulation via synthetic ligands makes NHRs of great biomedical interest, with vast current medical application and further untapped potential.

Molecularly, NHRs share common structural and functional features, including an N-terminal activation domain, a DNA binding domain (DBD), a hinge region, a ligand-binding domain (LBD), and an optional C-terminal F domain of unknown function (Sever and Glass, 2013; Weikum et al., 2018; Arao and Korach, 2021). The LBD binds ligands, whose presence or absence can regulate NHR activity, with additional regulation via post-translational modifications in the LBD and elsewhere (Berrabah et al., 2011). The LBD also serves as a binding site for transcriptional coregulators, including coactivators and corepressors, which influence NHR transcriptional output (Mouchiroud et al., 2014; Khan and Okafor, 2022; Scholtes and Giguère, 2022). The DBD and LBD also play roles in dimerization, as NHRs can function as monomers, homodimers, and/or heterodimers.

NHRs are conserved in metazoans, with high evolutionary conservation in the DBD and LBD. Interestingly, large expansions have led to speciation and divergence of NHRs in some animals. Notably, *Caenorhabditis elegans* encodes 284 NHRs, whereas humans encode 48, mice 49, and *Drosophila melanogaster* 18 (Magner and Antebi, 2008). Of the 284 *C. elegans* NHRs, 15 have clear homologs and play important roles in sex determination, development, molting, and aging; these include the best characterized NHR of *C. elegans*, DAF-12 (abnormal dauer formation), which regulates numerous developmental and physiological processes (Taubert et al., 2011; Hoffmann and Partridge, 2015; Kostrouchova and Kostrouch, 2015). The remaining 269 NHRs in *C. elegans* arose from duplications of an ancestral gene related to hepatocyte nuclear factor 4 alpha (HNF4α). HNF4α is a conserved NHR found in many species ranging from sponges to *D. melanogaster* to vertebrates (Sluder et al., 2002; Sladek, 2011; Taubert et al., 2011). Mammalian HNF4α regulates liver and pancreas development and function, and *D. melanogaster* HNF also controls lipid metabolism (Palanker et al., 2009; Kotulkar et al., 2023).

The expansion of the HNF4-related NHR family in *C. elegans* is interesting. Although the function of many of these NHRs is poorly understood, roles have emerged in the regulation of metabolism, stress adaptation, innate immune responses, and aging. NHR-80 is important in longevity (Goudeau et al., 2011; Folick et al., 2015). NHR-64, -66, and -80, function in lipid metabolism, and NHR-86 functions in lipid storage (Brock et al., 2006; Arda et al., 2010; Liang et al., 2010; Pathare et al., 2012). NHR-10, -68, and -114 function in vitamin B12-dependent metabolic pathways for the methionine/S-adenosylmethionine (Met/SAM) cycle and propionate breakdown (Gracida and Eckmann, 2013; Bulcha et al., 2019; Giese et al., 2020; Qin et al., 2022; Goh et al., 2023). Several NHRs are involved in pathogen defence, including NHR-14 and -86 in the response to *Pseudomonas aeruginosa* (Ward et al., 2014; Peterson et al., 2019; Rajan et al., 2019; Peterson et al., 2023), NHR-45 and -156 in the response to the mold *Penicillium brevicompactum* (Wallace et al., 2021), and NHR-42 after infection with *Staphylococcus aureus* (Goswamy et al., 2023). In addition, NHR-46 functions downstream of HIF-1, and is involved in the regulation of egg-laying and response to stress (Pender and Horvitz, 2018). However, the best-studied HNF4-like NHR in *C. elegans* is NHR-49 (Van Gilst et al., 2005a), which has emerged as an important regulator of lipid metabolism, stress responses, innate immune signalling, and lifespan, with roles in several cellular signalling pathways.

## 2 NHR-49 is an important regulator of lipid metabolism

NHR-49 is related to HNF4α based on sequence similarity, and *in silico* three-dimensional modeling analysis showed high-confidence resemblance when full-length NHR-49 was modeled onto a pre-existing HNF4α scaffold (Lee et al., 2016). Due to NHR-49’s role in fatty acid β-oxidation (see below), several reports have hypothesized that NHR-49 is a functional homolog of mammalian peroxisome proliferator-activated receptor alpha (PPARα) (Van Gilst et al., 2005a; Ratnappan et al., 2014). However, *in silico* modeling revealed a weaker similarity with PPARα (Lee et al., 2016). Interestingly, *C. elegans* lacks an apparent sequence homolog of PPARα. Therefore, although more closely resembling HNF4α in sequence and structure, NHR-49 may have evolved PPARα-like functions, or perhaps a combination of the functions of both proteins.

NHR-49 is thought to both homodimerize and heterodimerize with other transcription factors (Brelivet et al., 2004; Taubert et al., 2006; Pathare et al., 2012). For example, NHR-49 is thought to dimerize with NHR-80 to activate genes for fatty acid desaturation and dimerize with NHR-66 to repress genes involved in lipid remodeling (Pathare et al., 2012). NHR-49 also interacts with transcriptional coregulators, including the MDT-15 subunit of the Mediator complex (Taubert et al., 2006).

Its initial description and subsequent reports show that NHR-49 is an important regulator of *C. elegans* metabolism and lifespan. The allele used in the first study, *nr2041*, contains an 893 bp deletion spanning parts of the DBD and LBD and is a null mutant (Van Gilst et al., 2005a). Studies using this mutant as well as RNA interference (RNAi) showed that NHR-49 activates genes involved in lipid metabolism, especially fatty acid desaturation and mitochondrial fatty acid β-oxidation (Van Gilst et al., 2005a; Pathare et al., 2012; Ratnappan et al., 2014).

NHR-49 is an important regulator of mitochondrial β-oxidation, i.e., the breakdown of fatty acids to acetyl-CoA, which feed into the tricarboxylic acid (TCA) cycle to produce energy (Watts and Ristow, 2017; Adeva-Andany et al., 2019). NHR-49 activates the expression of important genes in this process, including *acs-2*, *cpt-5*, and *ech-1.1* (Van Gilst et al., 2005a). The acyl-CoA synthetase (ACS) *acs-2* functions in the first step of mitochondrial β-oxidation, where it activates fatty acids by catalyzing the binding of a CoA to form fatty acyl-CoA esters. Carnitine palmitoyltransferases (CPTs) such as *cpt-5* then transfer the acyl-CoA into the mitochondria, where it is broken down into acetyl-CoA by multiple enzymes including the enoyl-CoA hydratase *ech-1.1* (Adeva-Andany et al., 2019). Van Gilst et al. hypothesized that reduced expression of these genes in *nhr-49* null mutant worms accounts for the high-fat phenotype observed in L4 mutant worms using Nile Red stain, as over-expression of *acs-2* is sufficient to rescue fat levels back to wild-type (Van Gilst et al., 2005b). However, other studies describe an opposite effect in older adult *nhr-49* mutant worms, which display less fat than wild type, as determined by Oil Red O staining (Ratnappan et al., 2014; Watterson et al., 2022a). It is thus possible that age plays a role in the effect that NHR-49 has on lipid metabolism and fat storage.

NHR-49 also regulates fatty acid desaturation. NHR-49 activates the expression of the palmitoyl-CoA desaturase gene *fat-5*, whose product catalyzes the conversion of palmitic acid (C16:0) to palmitoleic acid (C16:1n7), and the stearoyl-CoA desaturases *fat-6* and *fat-7*, whose products catalyze the conversion of stearic acid (C18:0) to oleic acid (C18:1n9) (Van Gilst et al., 2005a). These desaturation reactions are not only important to maintain membrane fluidity and control lipid metabolism, but in *C. elegans*, the stearoyl-CoA desaturases also catalyze the first step in polyunsaturated fatty acid (PUFA) synthesis (Watts and Browse, 2002; Watts and Ristow, 2017). Thus, NHR-49 acts as an important modulator balancing lipid consumption and storage, likely in response to varying energy needs.

NHR-49 also regulates lipid metabolism via gene repression, including genes involved in sphingolipid metabolism and lipid catabolism. In this context, NHR-49 physically interacts with NHR-66 to repress genes such as the acid ceramidase *asah-2*, the sphingosine-phosphate lyase *spl-2*, the lipase *lips-6*, and the O-acyltransferase *oac-56* (Pathare et al., 2012). In sum, NHR-49 controls several aspects of *C. elegans* lipid metabolism, likely partnering with other NHRs to achieve specific regulation.

## 3 NHR-49 is required for several different stress responses

**FIGURE 1:**
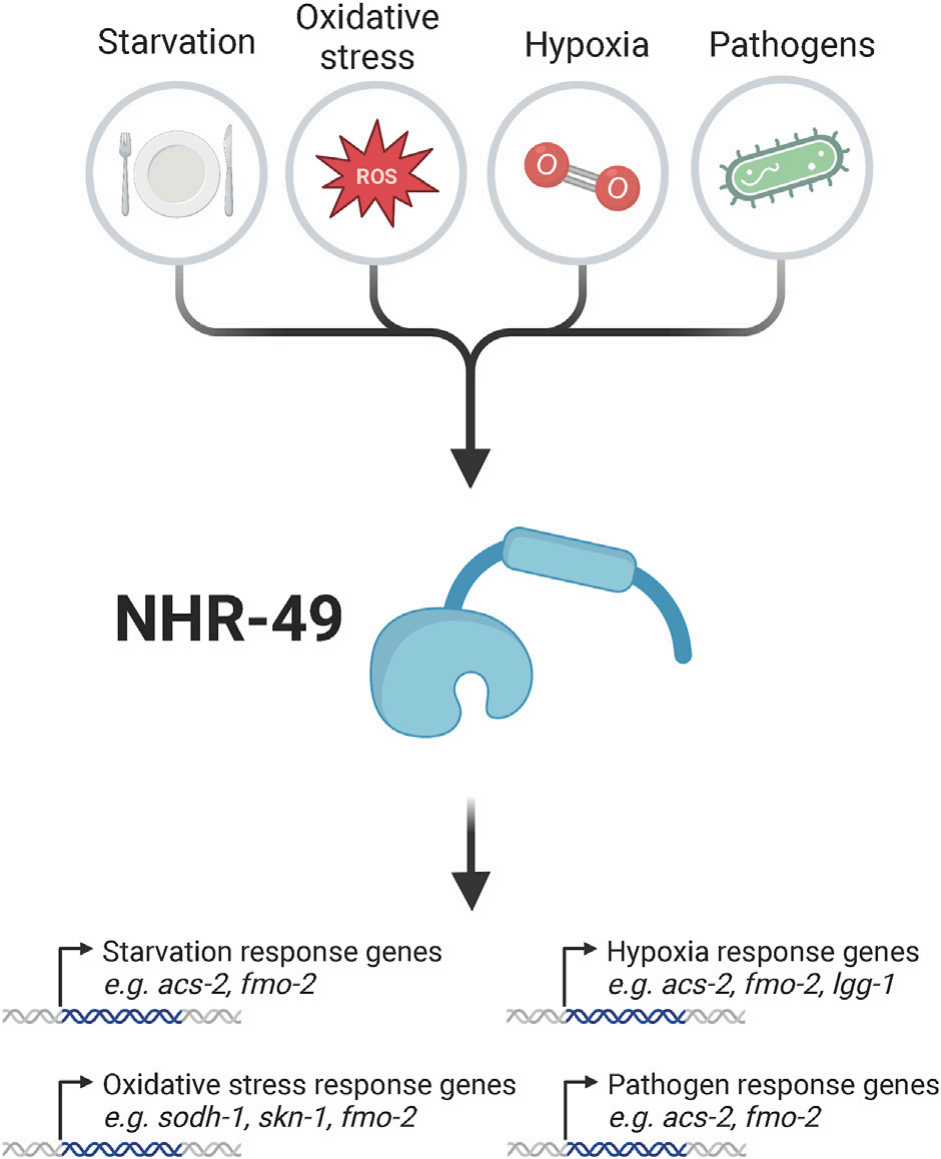
Overview of exogenous stressors which activate NHR-49 to promote the activation of stress response genes. Known stressors that influence NHR-49 activity include starvation, oxidative stress, hypoxia, and infection of *Caenorhabditis elegans* with various pathogens. These stressors are sensed via unknown mechanisms by NHR-49, which in turn upregulates genes involved in stress regulation, such as *acs-2* and *fmo-2* in the starvation response; *sodh-1*, *skn-1*, and *fmo-2* in the oxidative stress response; *acs-2*, *fmo-2*, and *lgg-1* in the hypoxia response; and *acs-2* and *fmo-2* upon pathogen infection; at least some of these genes then provide resistance to the pertinent stresses. For details see text. Created with BioRender.com.

The role of NHR-49 in lipid metabolism is viewed as its primary role in the control of development and physiology. However, over the last few years, an additional important function as a regulator of stress responses has emerged for NHR-49. While likely at least in part linked to its effects on lipid biology, NHR-49 appears to regulate at least some separate genes and processes to directly control stress responses (Figure 1).

Cellular stresses are harmful insults of physical, chemical, or biological nature. Organisms are constantly exposed to endogenous and exogenous stresses. Thus, an organism’s ability to mount specific stress responses is critical to maintain cellular and organismal homeostasis. Initially, this response aims to protect healthy cells and tissues from harm by defending against and adapting to the insult. This usually involves signal transduction cascades, often leading to the activation of transcription factors that alter the expression of response genes to re-establish homeostasis. However, when damage to a cell cannot be overcome, cellular death programs such as apoptosis, necrosis, or autophagy-induced cell death are activated to eliminate damaged cells (Fulda et al., 2010; Galluzzi et al., 2018). NHR-49 has emerged as an important factor in stress responses of *C. elegans*, and this relatively new role of NHR-49 is reviewed below.

### 3.1 NHR-49 in the starvation response

Starvation is defined as the short or long-term absence of nutrients, including caloric energy, below the threshold that is needed to support the life of an organism (Baugh and Hu, 2020). In its natural habitat, *C. elegans* leads a boom-or-bust lifestyle characterized by a prolific and rapid reproduction, typically experiencing either plentiful food environments or else a complete absence of food. Related laboratory experiments usually mimic these conditions, providing either food *ad libitum* or not at all (note, dietary restriction is discussed below).

*C. elegans* features several responses to starvation that involve different regulatory factors and that lead to distinct outcomes for the animal depending on its developmental stage at the onset of starvation. These adaptations include the dauer diapause, the adult reproductive diapause (ARD), and larval arrest [for details, see (Baugh and Hu, 2020)]. For example, the response to larval stage 1 (L1) starvation involves rewiring of energy metabolism from anabolic processes towards catabolic lipolysis. This is regulated by the transcription factor helix-loop-helix 30 (HLH-30), the homolog of the mammalian transcription factor EB (TFEB) (O’Rourke and Ruvkun, 2013; Settembre et al., 2013).

NHR-49 is an important regulator of the starvation response, with roles in several of the aforementioned stages. When food is available, *nhr-49* is required for the expression of many lipid metabolism genes, including *fat-7* and *acs-2* (Van Gilst et al., 2005a; Van Gilst et al., 2005b)*.* However, when nutrients are limited in L1 or L4 stage larvae, animals break down fats stored in lipid droplets to satisfy their energy requirements, and NHR-49 plays a key role in controlling this process. Specifically, following a 12 h starvation, *acs-2* is highly induced by NHR-49, whereas expression of *fat-7* is downregulated across all worm stages (Taubert et al., 2006; Van Gilst et al., 2005b). *nhr-49* is also required to induce non-lipid metabolism related genes, including the glyoxylate cycle enzyme gene *icl-1*, the oxidoreductase gene *sodh-1*, and the flavin-containing monooxygenase gene *fmo-2* (Goh et al., 2018). Although it is not clear if or how these genes all contribute to metabolic remodeling in starvation, *fmo-2* is required in wild-type worms for starvation survival (Goh et al., 2018). In addition, NHR-49 acts in the intestine during short-term starvation (2 h) to limit lipid accumulation within lysosomes. NHR-49 does so by upregulating lysosomal hydrolases and phospholipases to catabolize lipids within this organelle (Huang et al., 2014). Due to its important downstream regulatory functions in starvation, *nhr-49* is thus required for L1 stage starvation recovery in *C. elegans* (Goh et al., 2018).

NHR-49’s relationship with other starvation response regulators suggests nonredundant functions. HLH-30 is a master regulator of the starvation response that regulates lysosome biogenesis and autophagy (Lapierre et al., 2013; Settembre et al., 2013; Harvald et al., 2017). In the mammalian liver, its ortholog TFEB controls expression of the PPARα–PPARγ coactivator 1 alpha (PGC-1α) complex to regulate lipid metabolism during starvation (Settembre et al., 2013). However, no data to date support a similar interaction between HLH-30 and NHR-49 in *C. elegans*. In fact, HLH-30 seems to be dispensable or only partially required for the induction of *nhr-49*-dependent stress response genes (Leiser et al., 2015; Goh et al., 2018). This, along with the synthetic lethality seen in the attempt to make an *nhr-49;hlh-30* double null mutant (Goh et al., 2018) suggests that these two factors act non-redundantly in *C. elegans*.

To regulate transcription of target genes, NHR-49 must localize to the nucleus. Watterson et al. observed this under starved conditions by studying animals that overexpress an NHR-49::GFP fusion protein (Watterson et al., 2022b). In *ad libitum* fed conditions, NHR-49 is bound to cytosolic vesicles by the small G protein RAB-11.1 (Watterson et al., 2022b). However, during starvation, loss of lipid homeostasis causes NHR-49 to be released from these vesicles, whereupon it localizes to the nucleus to activate genes such as *acs-2* (Watterson et al., 2022b). Interestingly, NHR-49’s release from endocytic vesicles can also be triggered by loss of the heat shock factor *hsf-1*, which leads to increased NHR-49 nuclear localization and activity (Brunquell et al., 2016; Watterson et al., 2022a). Future work into this mechanism could distinguish if NHR-49 nuclear localization is controlled by similar mechanisms in other stresses to which NHR-49 provides functional adaptation (see below).

When L3 or L4 stage worms are deprived of food, they arrest in a specialized adult stage termed the ARD. The germline of these animals contains only a few quiescent stem cells. This allows the worms to extend their lifespan 3-fold, providing an opportunity to locate food (Angelo and Van Gilst, 2009). When food is available, these worms can recover and produce progeny as normal (Angelo and Van Gilst, 2009; Baugh and Hu, 2020; Gerisch et al., 2020). Although *nhr-49* is not involved in ARD when beginning the starvation period during the mid-L3 stage (Gerisch et al., 2020), starvation onset in mid-L4 worms requires *nhr-49* for entry into and recovery from adult reproductive diapause (Angelo and Van Gilst, 2009; Eustice et al., 2022). *nhr-49* may be important for entry into ARD due to its requirement in inducing β-oxidation (Eustice et al., 2022). Thus, NHR-49 plays a major role in controlling the cellular response to several different starvation responses, likely acting both through lipid metabolism and other genes and processes.

### 3.2 NHR-49 in the oxidative stress response

Oxidative stress occurs when reactive oxygen species (ROS) accumulate within the cell to toxic levels. ROS are produced endogenously as obligate and ubiquitous by-products of aerobic respiration, mainly by electron transport chain (ETC) complexes I and III, when leaky electrons form superoxide. Substantial amounts of ROS are produced by peroxisomes and by enzymes such as cytochrome P450 and NADPH oxidases (Halliwell, 1991; Lenaz, 2001; Shields et al., 2021; Sies et al., 2022). ROS can also be taken up exogenously or produced by the environment. For example, ROS levels increase as a consequence of exposure to heavy metals, xenobiotics, radiation such as UV-C, and other sources. In these contexts, ROS can cause transient or irreversible damage to macromolecules such as DNA, RNA, lipids, including membrane lipids, and proteins (Shields et al., 2021; Sies et al., 2022). Cellular systems have evolved to neutralize and limit ROS accumulation, including non-enzymatic molecules glutathione and flavonoids, and enzymes such as superoxide dismutases (SOD) and catalases (Martindale and Holbrook, 2002). However, at controlled levels, ROS are important for physiological functions such as innate immune responses, development, and cytoskeletal organization (Wilson and González-Billault, 2015; Miranda-Vizuete and Veal, 2017; Oswald et al., 2018; Wilson et al., 2018; Sies and Jones, 2020; Al-Shehri, 2021). Homeostasis is achieved when the generation and removal of ROS is properly controlled, ensuring cellular function while avoiding or faithfully repairing damage caused by ROS.

The evolutionarily conserved Cap “n” collar (CNC)-basic leucine zipper (bZIP) transcription factor Nrf2, encoded by the nuclear factor, erythroid-derived 2-like 2 (NFE2L2) gene, is often considered a master regulator of oxidative stress responses (Alam et al., 1999; Ma, 2013; Blackwell et al., 2015). In *C. elegans*, oxidative stress responses typically require skinhead (*skn-1*), the homolog of Nrf2 (An and Blackwell, 2003; Blackwell et al., 2015). Indeed, *skn-1* is vital for animal survival after exposure to many oxidative stressors, including paraquat, sodium arsenite, and tert-butyl hydroperoxide (tBOOH). SKN-1 is also an important regulator of longevity (An and Blackwell, 2003; An et al., 2005; Tullet et al., 2008; Oliveira et al., 2009; Blackwell et al., 2015; Steinbaugh et al., 2015).

Interestingly, however, the transcriptional response to one oxidative stressor, tBOOH, is at least partially independent of SKN-1 (Oliveira et al., 2009; Goh et al., 2014). A substantial part of the response to tBOOH instead requires *nhr-49* and its coregulator *mdt-15* (Goh et al., 2014; Goh et al., 2018). Consequently, loss of *nhr-49* renders worms sensitive to tBOOH; in addition, loss of *nhr-49* sensitizes worms to arsenite and paraquat (Horikawa and Sakamoto, 2009; Goh et al., 2014; Goh et al., 2018), although *nhr-49*’s involvement in transcriptomic changes caused by these molecules has not yet been defined. In the tBOOH response, *nhr-49* is required for the upregulation of dozens of genes, including *fmo-2*, *dhs-18*, *icl-1*, *sodh-1*, and *nlp-25*, several of which NHR-49 also induces during starvation (Goh et al., 2018). Knockdown of the oxidoreductase *sodh-1* and K05B2.4, predicted to encode an enzyme with acyl-CoA hydrolase activity, rendered worms sensitive to tBOOH, suggesting that activation of these enzymes may be how NHR-49 promotes oxidative stress protection. In contrast, loss of *fmo-2*, a gene highly induced by tBOOH and completely dependent on *nhr-49* for induction, paradoxically increased worm survival in this context (Goh et al., 2018), perhaps because FMO-2’s predicted oxidase activity leads to ROS production, exacerbating the stress.

Interestingly, NHR-49 appears to be in a regulatory relationship with SKN-1. Specifically, *nhr-49* gain of function mutant strains showed induction of the Glutathione S-Transferase *gst-4*, a highly stress sensitive gene that requires *skn-1* for activation in most contexts (An and Blackwell, 2003). Indeed, like *skn-1*, *nhr-49* and *mdt-15* are required for *gst-4* induction in response to arsenite and paraquat (Hu et al., 2018), although *nhr-49* was dispensable to induce several SKN-1-induced genes, including *gst-4*, in another study (Goh et al., 2018). Nevertheless, *nhr-49* is needed to activate the expression of the SKN-1c isoform, the key isoform driving antioxidant responses, in a worm strain mutant for *brap-2/BRCA1 Associated Protein homolog*, and is essential for increased SKN-1 activity in the *amdh-1/*aminohydrolase domain containing protein mutant, although it is not clear if this is a transcriptional or posttranscriptional role of NHR-49 (Hu et al., 2018; Frankino et al., 2022). Another link between these two regulators is that they share MDT-15 as a physical interactor and functional coregulator (Taubert et al., 2006; Goh et al., 2014). Together, these studies suggest that the SKN-1 and NHR-49 pathways for oxidative stress resistance crosstalk, possibly to achieve optimal transcriptional response to pro-oxidant conditions.

NHR-49 and MDT-15 also regulate peroxisomal quality control genes and some genes involved in peroxisomal β-oxidation (Van Gilst et al., 2005a; Rackles et al., 2021). Thus, although *nhr-49* promotes the oxidation of fatty acids in peroxisomes and mitochondria, processes which generate ROS, it also has an oxidoprotective role for organismal survival during oxidative stress. Perhaps these activities are coordinated, such that when NHR-49 induces fatty acid oxidation for energy production, it induces genes such as *sodh-1* and *skn-1* that protect against the concurrently produced ROS*.*

### 3.3 NHR-49 in the hypoxia response

Hypoxia is a stress that occurs when cellular oxygen levels are too low for normal physiological functions. It occurs naturally in cells and tissues during development, as well as in many diseases (Powell-Coffman, 2010; Lee et al., 2020). Aerobic respiration, the principal source of energy generation in most eukaryotes, requires oxygen. As a small organism without a dedicated respiratory system, *C. elegans* receives oxygen in all cells of its body by diffusion. In animals, cellular damage and death through apoptosis can occur when oxygen availability drops below the physiologically required level (Carreau et al., 2011). Thus, adaptation to hypoxia is critical for maintaining cellular and organismal health.

The pathways that regulate the response to hypoxia are evolutionarily conserved. As in mammals, a key pathway in *C. elegans* involves the transcription factor hypoxia inducible factor 1 (HIF-1; the sole *C. elegans* homolog of mammalian HIF*a*), which is critical for the cellular responses to and the defence against hypoxia (Jiang et al., 2001; Choudhry and Harris, 2018). To survive hypoxia (0.3%–1% O_2_ in *C. elegans*) (Jiang et al., 2001), worms activate the EGL-Nine homolog (*egl-9*)–von Hippel–Lindau (*vhl-1)*–*hif-1* pathway. In normoxic conditions (21% O_2_), HIF-1 is degraded and thus inactive. This occurs when EGL-9 adds a hydroxyl group onto a proline residue in HIF-1. The hydroxylated proline promotes binding of the E3 ubiquitin ligase VHL-1 (the *C. elegans* VHL homolog), leading to poly-ubiquitination and proteasomal degradation of HIF-1. However, in hypoxic conditions, EGL-9 hydroxylation is rendered inactive by the lack of oxygen; hence, HIF-1 is stabilized, allowing it to dimerize with the HIF1b homolog AHA-1 and to activate a hypoxia adaptation gene program (Epstein et al., 2001; Powell-Coffman, 2010). Accordingly, *C. elegans* carrying *vhl-1* or *egl-9* mutations show increased HIF-1 protein levels in normoxic conditions (Epstein et al., 2001), and loss of *hif-1* renders worms sensitive to hypoxic exposure (Jiang et al., 2001; Shen et al., 2005).

In addition to the HIF-1 responses, several parallel transcriptional programs exist in *C. elegans* and mammalian organisms that are critical for protection from hypoxia (Pursiheimo et al., 2009; Li et al., 2013; Padmanabha et al., 2015; Valko et al., 2021). NHR-49 is an important contributor to hypoxia resistance in *C. elegans*, as its loss results in hypoxia sensitivity that is equivalent to that caused by loss of *hif-1*. Concomitant loss of both genes results in virtually complete lethality in hypoxia, demonstrating that *nhr-49* and *hif-1* act non-redundantly and in separate pathways (Doering et al., 2022).

How does *nhr-49* promote protection from hypoxia? During hypoxia, damaged cellular components can be cleared or recycled via autophagy (Mazure and Pouysségur, 2010; Tan et al., 2016). *C. elegans* show sensitivity to hypoxia and anoxia when the autophagy pathway is disrupted (Samokhvalov et al., 2008; Doering et al., 2022), and autophagy genes as well as the formation of autophagosomes are upregulated in hypoxia and anoxia (Chapin et al., 2015; Doering et al., 2022). Critically, *nhr-49* is required to upregulate both autophagy genes and autophagosome formation in hypoxia, and autophagy genes act in the same pathway, and independently of *hif-1*, to promote survival in hypoxia.

Activation of autophagy appears to be a key function of NHR-49 driven hypoxia adaptation but is likely not the only one. In hypoxia, NHR-49 also induces a suite of detoxification genes (Doering et al., 2022). Interestingly, a separate set of detoxification genes depend only on *hif-1*, and a third set of detoxification genes is independent of both *nhr-49* and *hif-1*. This suggests that induction of detoxification genes in hypoxia is an important process that is achieved by multiple transcription factors acting in parallel in hypoxia. Autophagy and detoxification are also enriched biological processes dependent on *nhr-49* for induction in oxidative stress caused by tBOOH (Goh et al., 2018). However, *nhr-49* regulates some unique sets of genes involved in each process in each stress, suggesting some genes and processes regulated by *nhr-49* are stress-specific, whereas some are common amongst stresses (Doering et al., 2022).

Another important role of NHR-49 in hypoxia relates to extracellular matrix remodelling. In *C. elegans*, hypoxia results in cuticle disorganization. The hypoxia inhibited receptor tyrosine kinase HIR-1 coordinates remodelling of the extracellular matrix, and the downstream signalling pathway is HIF-1-independent but linked to NHR-49. Although *nhr-49* loss does not affect cuticle organization, *nhr-49* is required to regulate the expression of many cuticle-related genes in the *hir-1* mutant (Vozdek et al., 2018). NHR-49’s role in hypoxia resistance therefore may include physiological and developmental adaptations.

As a nuclear receptor, NHR-49 likely functions in the nucleus to regulate gene expression, and some NHRs can enter the nucleus when bound to their cognate ligand. However, this may not be the case for NHR-49 in hypoxia, as Vozdek et al. found that the subcellular localization of a NHR-49::Venus fusion protein did not change after hypoxia exposure (Vozdek et al., 2018). Doering et al. did not observe a change in localization during hypoxia, either, but found that an overexpressed, fluorescently tagged NHR-49::GFP fusion protein was mildly induced by hypoxia (Vozdek et al., 2018; Doering et al., 2022).

Changes in overall levels and/or subcellular localization of transcription factors is often governed by upstream kinases via phosphorylation. Although no kinase has yet been shown to directly target NHR-49, evidence for such regulation has begun to emerge. Homeodomain-interacting protein kinase-1 (HPK-1), the only *C. elegans* HIPK homolog, is a nuclear kinase that participates in stress response (Rinaldo et al., 2007; Berber et al., 2013; Berber et al., 2016; Das et al., 2017). *nhr-49* coordinates the hypoxia response with *hpk-1*, which is required to promote accumulation of NHR-49 in low oxygen and to induce autophagy genes and autophagosome formation (Doering et al., 2022). HPK-1 also regulates autophagosome formation during dietary restriction (Das et al., 2017), another context regulated by NHR-49 (see below). Future work could determine if these two factors work together in the response to additional stresses and whether NHR-49 is a direct target of HPK-1 kinase activity.

To date, *nhr-49*’s role in hypoxia response has been studied in the HIF-1-depedenent range of oxygen concentration (i.e., 1%–0.3% O_2_). However, the responses to severe hypoxia (<0.3%) and anoxia (0%) involve additional, *hif-1*-independent pathways (Powell-Coffman, 2010). Whether *nhr-49* is essential in these conditions is unknown. In 0.5% oxygen, RNA-sequencing analysis showed that approximately 26% (83 of 315) of *nhr-49*-dependent genes are *hif-1*-independent, including autophagy genes and detoxification genes (Doering et al., 2022). This may suggest that *nhr-49*, having some *hif-1*-independent functions at 0.5% oxygen, may also regulate these genes in 0.1% or 0% oxygen. In particular, autophagy genes are essential for survival in anoxia (Samokhvalov et al., 2008), so *nhr-49* may be required in these conditions to promote survival via autophagy. Further research into the role of NHR-49 in hypoxia adaption will be interesting.

### 3.4 NHR-49 in the heat shock response

A recent report revealed that NHR-49 may also play a role in the heat shock and proteostasis response of *C. elegans* (Sala et al., 2023). Specifically, although *nhr-49* loss did not render wild-type background animals sensitive to acute heat shock, it did cause such sensitivity in several contexts that feature enhanced thermotolerance. Moreover, the *nhr-49(et7)* gain of function mutation promoted thermotolerance, which required the conserved *hsf-1* master regulator. Mechanistically, *nhr-49* gain caused the activation of heat shock protein family chaperones. This interplay of NHR-49 with HSF-1 resembles that of NHR-49 with SKN-1 in some oxidative stress responses and generally suggests that NHR-49’s interactions with other critical stress response pathways may reveal interesting regulatory paradigms.

### 3.5 NHR-49 in innate immune responses

In their natural habitat, *C. elegans* encounters many pathogens. While *C. elegans* lacks an adaptive immune system and mobile immune cells, it has an innate immune system to activate sophisticated responses to escape from or resist and survive infection (Ermolaeva and Schumacher, 2014; Kim and Ewbank, 2018; Martineau et al., 2021; Tran and Luallen, 2023). Innate immunity is the first line of protection against pathogens and refers to the non-specific defense systems. As a bacterivore, *C. elegans* is an excellent model to study the process of and the response to infection, allowing for *in vivo* study of pathogenesis and response to intestinal infections simply via feeding. Interestingly, upon infection with common human pathogenic bacteria such as *P. aeruginosa*, *S. aureus*, and *Enterococcus faecalis*, many upregulated genes including *icl-1*, *fmo-2*, and *acs-2*, are regulated by NHR-49 (Dasgupta et al., 2020; Naim et al., 2021; Wani et al., 2021), revealing a role for this transcription factor in the innate immune response.

The round-shaped, Gram-negative bacterium *P. aeruginosa* colonizes the *C. elegans* intestine. Here, infection causes virulence-related membrane vesicles to accumulate and forms an extracellular biofilm matrix similar to infection in the mammalian lung. As the infection progresses, the intestine becomes distended, intracellular invasion begins, and abnormal autophagosomes form, eventually leading to animal death (Powell and Ausubel, 2008; Irazoqui et al., 2010). The Gram-positive bacteria *S. aureus* and *E. faecalis* also colonize the intestine of *C. elegans* (Irazoqui et al., 2010). Although both bacteria cause intestinal distention, *S. aureus* infection results in thinning and destruction of the intestine, anal deformations, and degradation of other organs in the worm (Garsin et al., 2001; Irazoqui et al., 2010).

*nhr-49* is required for *C. elegans* survival upon infection with *P. aeruginosa*, *S. aureus*, and *E. faecalis* (Sim and Hibberd, 2016; Dasgupta et al., 2020; Naim et al., 2021; Wani et al., 2021); similarly, the compound LK56, which stimulates innate immunity, uses NHR-49 and its coregulator MDT-15 to confer resistance to these bacteria (Hummell et al., 2021). Although the intestine is the main organ affected by infection with the aforementioned bacteria, the tissue-specific roles NHR-49 plays in response to each bacteria vary. Although re-expression of NHR-49 in any of the intestine, neuron, muscle, or hypodermis is sufficient to rescue the infection survival defect of *nhr-49* mutant worms on *P. aeruginosa*, NHR-49 expression only in the intestine or neurons rescues survival back to wild-type levels after infection with *S. aureus* (Naim et al., 2021; Wani et al., 2021). Additionally, overexpressing NHR-49 in the neurons enhances resistance during *E. faecalis* infection compared to wild type, whereas intestinal overexpression has no effect on animal survival (Dasgupta et al., 2020). This suggests that, like in other stresses, NHR-49 can regulate *C. elegans* innate immune responses in both cell autonomous and cell non-autonomous fashions.

How does NHR-49 promote innate immune responses? Work to date has largely focused on two NHR-49 regulated genes, *acs-2* and *fmo-2*, and has suggested an immunometabolic role for NHR-49. *acs-2* is induced during *E. faecalis* and *P. aeruginosa* infection but is only required for worm survival after infection with *E. faecalis* (Dasgupta et al., 2020; Naim et al., 2021). *fmo-2* is induced by and required for survival after infection with *S. aureus* and *E. faecalis*, but is downregulated after infection with *P. aeruginosa* (Dasgupta et al., 2020; Naim et al., 2021; Wani et al., 2021). *nhr-49* is also required to induce some C-type lectin genes in response to *S. aureus* infection (Wani et al., 2021). C-type lectins, which can bind to carbohydrates and pathogens, are another group of genes with roles in immune response in vertebrates and invertebrates. The *C. elegans* genome encodes 283 C-type lectins, many of which are induced by only one or two specific pathogens, highlighting a potential role in immune response specificity (Schulenburg et al., 2008; Pees et al., 2021). Future work could determine if NHR-49 regulates different subsets of C-type lectin genes in response to distinct pathogens, and the roles they may play. Additional genes that are induced by NHR-49 during infection and require further investigation into their immune-specific role include *icl-1* and the lipase *lipl-3* on *E. faecalis*, and *fmo-5* in *S. aureus* (Dasgupta et al., 2020; Wani et al., 2021). This highlights that *C. elegans* features pathogen-specific responses and suggests that NHR-49 regulates different subsets of response genes depending on which pathogen it is infected by.

Although NHR-49 and HLH-30 act in parallel pathways during starvation and lifespan (Goh et al., 2018; Wani et al., 2021), an interesting connection between these transcription factors exists in innate immune responses. *hlh-30* is required to express many genes following infection with *S. aureus*, but a subset of infection-response genes is *hlh-30*-independent, and instead requires *nhr-49* for induction (Visvikis et al., 2014; Wani et al., 2021). Interestingly, NHR-49 and HLH-30 contribute to each other’s expression in response to *S. aureus* infection and thus may cooperate, perhaps to achieve optimal response after infection (Wani et al., 2021). Furthermore NHR-80, which can dimerize with NHR-49 (Pathare et al., 2012), is also required for *C. elegans* survival after infection with *P. aeruginosa* (Naim et al., 2021). Future work could determine if these two factors function together, potentially as dimerization partners, to control the innate immune response.

Although much of the work on NHR-49’s role in innate immunity has focused on bacterial pathogens, *C. elegans* are infected by many microorganisms. Examples include the fungal pathogens *Drechmeria coniospora* and *Harposporium* sp., the microsporidia genus *Nematocida,* and the Orsay virus (Gammon, 2017; Schulenburg and Félix, 2017). Future work may determine if the immunometabolic role NHR-49 plays may be broader and not limited to bacterial infection.

## 4 NHR-49 in the regulation of life and healthspan

### 4.1 NHR-49 is an important regulator of lifespan in wild-type *Caenorhabditis elegans*

NHR-49 plays an important role in regulating the lifespan of *C. elegans*. At 20°C, a common temperature to cultivate *C. elegans* in the laboratory, *nhr-49* loss shortens lifespan, whereas *nhr-49* gain (overexpression) extends lifespan in wild type. In loss of function studies, the commonly used *nhr-49* deletion allele, *nr2041*, decreases the mean lifespan of wild-type animals from 20 days to approximately 14–15 days at 20°C (Van Gilst et al., 2005a; Ratnappan et al., 2014). Another deletion allele, *gk405*, shortens lifespan to a similar extent (Lee et al., 2019). RNAi knockdown of *nhr-49* also reduces the lifespan of wild-type worms, albeit to a lesser extent than *nhr-49* mutation (Van Gilst et al., 2005a; Khan et al., 2013). Interestingly, the effect of *nhr-49* loss on wild-type lifespan is temperature-dependent, as the *nhr-49(gk405)* mutant shows a wild-type lifespan at 25°C, but a substantially reduced lifespan at 15°C, which extends worm lifespan compared to 20°C and 25°C (Lee et al., 2019). Conversely, overexpressing NHR-49 from its own promoter rescues the short lifespan of the *nhr-49* null mutant, and, importantly, increases the lifespan of wild-type worms to a mean of 26 days at 20°C (Ratnappan et al., 2014), demonstrating sufficiency. More ambiguous are the effects of the *nhr-49* gain of function alleles, *nhr-49(et7) (P479L)*, *nhr-49(et8) (S432F)*, and *nhr-49(et13) (V411E)*. Specifically, *et7* increases lifespan, *et8* decreases lifespan, and *et13* has no effects (Lee et al., 2019). The reason for this discrepancy is unclear; these alleles were identified in a screen for mutants that suppress the cold sensitivity caused by the loss of the mammalian adiponectin transmembrane receptor homolog *paqr-2* (Svensk et al., 2013), not for lifespan related phenotypes. However, the *paqr-2–nhr-49* axis is important for longevity in some contexts such as dietary restriction ((Jeong et al., 2023); see below). Determining how *et7* promotes lifespan extension would be an interesting future research direction.

#### 4.1.1 NHR-49 has tissue specific roles in regulating longevity

As noted above, when expressed from its own promoter, *nhr-49* is sufficient to extend lifespan, and such overexpression also rescues the short lifespan of *nhr-49(nr2041)* mutants (Ratnappan et al., 2014). NHR-49 is expressed widely in *C. elegans* tissues, including the hypodermis, intestine, body wall muscle, neurons, and pharynx (Van Gilst et al., 2005a), and it is therefore of interest to understand in which tissue(s) it acts to regulate lifespan. Tissue-specific expression analysis revealed that NHR-49 expression in the neurons, intestine, or hypodermis can rescue the short lifespan of *nhr-49* mutants, whereas expression in the muscle does not (Naim et al., 2021). Interestingly, neuronal-specific NHR-49 overexpression extends lifespan beyond that of wild type, whereas intestinal overexpression does not (Burkewitz et al., 2015). In addition, neuron-specific *nhr-49* RNAi partially reduces lifespan extension induced by glucose restriction, whereas intestinal RNAi has no effect, suggesting that intestinal NHR-49 is dispensable (Jeong et al., 2023). Correspondingly, re-expressing NHR-49 in neurons or intestine of *nhr49(nr2041)* mutants rescues lifespan extension in glucose-restricted *C. elegans,* with a more pronounced effect in neurons (Jeong et al., 2023). Restoring NHR-49 expression in neurons is also sufficient for the lifespan extension achieved by activated AMP-activated protein kinase (AMPK), and to activate gene expression of NHR-49 regulated genes in the intestine (Burkewitz et al., 2015); we note that this study used the *rab-3* promoter, which was later found to have leaky expression in the intestine (Zhang et al., 2022), to drive neuronal NHR-49; however, a role for neuronally expressed NHR-49 in longevity is also supported by Naim et al., 2021. A role for neuronal NHR-49 in longevity is supported by tissue-specific rescue studies of lifespan in a double mutant of *nhr-49* with the long-lived, germline less *glp-1/Notch receptor* mutant: although expression of NHR-49 from intestine, hypodermis, and body wall muscle specific promoters all partially rescued *glp-1* longevity, expressing NHR-49 pan-neuronally completely restored long lifespan (Naim et al., 2021). Overall, these studies show that NHR-49 acts in several tissues to regulate longevity, but pinpoint function in neurons as especially critical. In the future, it would be interesting to define which genes NHR-49 regulates in neurons in longevity contexts.

#### 4.1.2 Role of NHR-49 in protection against proteotoxicity

Many factors that extend the lifespan of *C. elegans* also extend its healthspan, defined as healthy productive time before age-associated decline, as measured by phenotypes such as movement defects (Tissenbaum, 2012). One measure of healthspan is the ability of *C. elegans* to withstand proteotoxicity as caused by the expression of aggregation-prone transgenes. Loss of *nhr-49* function causes increased toxicity of an Aβ1-42 transgene mimicking aspects of Alzheimer’s disease, whereas NHR-49 overexpression increases resistance to Aβ1-42-induced toxicity (Leiteritz et al., 2020). Furthermore, the *nhr-49(gk405)* null allele displayed age-depended paralysis in Aβ1-42 transgenic *C. elegans* (Lee et al., 2019). In this context, as well as elsewhere, NHR-49 regulates the expression of genes involved in lipid metabolism and mitochondrial function, suggesting a mechanism for its protective effects against Aβ1-42 toxicity (Leiteritz et al., 2020). Similarly, increased NHR-49 activity via *nhr-49(et7)* mutation is sufficient to reduce the aggregation of a transgenic polyglutamine peptide, another model of an aggregation-prone age-related neurodegenerative disease; in this context, NHR-49 likely acts both via lipid metabolism as well as by promoting the expression of chaperones via HSF-1 (Sala et al., 2023), which may help restore or remove protein aggregates.

### 4.2 *nhr-49* is required for lifespan extension in several long-lived contexts

#### 4.2.1 *nhr-49* is a key effector in the long-lived *glp-1* mutant

**FIGURE 2:**
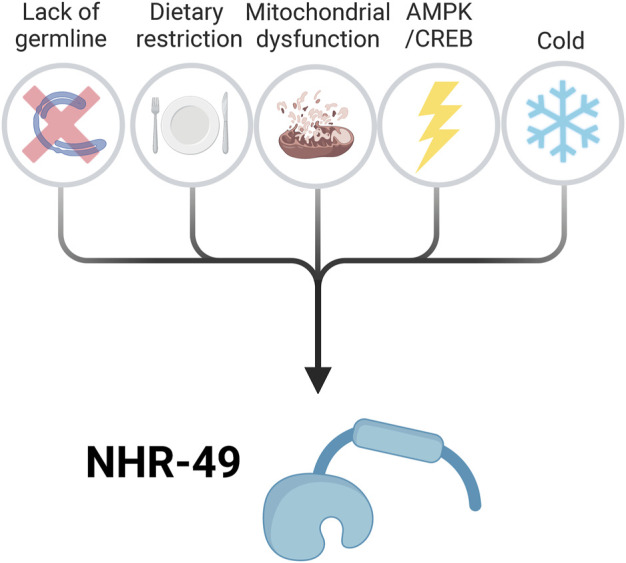
Overview of the signalling pathways that activate NHR-49 to promote lifespan extension. Known upstream signalling pathways and processes that influence NHR-49 activity include long-lived germline-less mutants, dietary restriction, mitochondrial dysfunction, activation of the AMPK/CREB pathway, and low temperature. These conditions induce *Caenorhabditis elegans* longevity at least in part by activating NHR-49, which promotes the expression of pro-longevity genes (see Figure 3). For details see text. Created with BioRender.com.

Besides its role in maintaining lifespan in wild-type worms, *nhr-49* is required to extend lifespan in many long-lived contexts, including genetic mutants and dietary conditions (Figure 2). One of the former is the germline-less *glp-1* strain, which carries a mutation in a notch receptor family member that is required for germline proliferation (Austin and Kimble, 1987). *glp-1* mutants lack a germline and are substantially long lived. Among other factors, the transcriptional regulators *daf-16*, a key longevity effector in many contexts, and transcription elongation regulator homolog *tcer-1* are required for the lifespan extension of *glp-1* mutants (Berman and Kenyon, 2006). *glp-1* longevity also requires *nhr-49*, and *nhr-49* mRNA is upregulated in somatic cells upon germline removal (Ratnappan et al., 2014). Mechanistically, *nhr-49* is a downstream target of DAF-16 and TCER-1 (Ratnappan et al., 2014). In contrast, in fertile adults with a healthy germline, *nhr-49* expression does not require *daf-16* and *tcer-1* (Ratnappan et al., 2014). Upregulation of *nhr-49* therefore appears to be a specific output of GLP-1–DAF-16–TCER-1 pro-longevity signaling.

*nhr-49* is also regulated by lysosomal lipid signaling in the *glp-1* context. This signaling originates in germline stem cells that upregulate lipid hydrolysis, mobilizing fat stores and contributing to healthy, long-lived *C. elegans* (Wang et al., 2008)*.* The lysosomal acid lipase LIPL-4 plays a key role, breaking down lipids, which can be bound by lipid chaperones LBP-8 and LBP-3 (Folick et al., 2015; Savini et al., 2022). LBP-8 acts in lysosome-to-nucleus signaling, whereas LBP-3 promotes fat-to-neuron signaling (Savini et al., 2022). These lipid transporters relay signals such as fatty acid ligands to NHR-80, a binding partner of NHR-49, which then increases longevity by modulating downstream gene expression (Pathare et al., 2012; Folick et al., 2015). Fluorescence and protease sensitivity assays show that oleoylethanolamide, a PPARα agonist (Fu et al., 2003), serves as a direct ligand for NHR-80, but not NHR-49 (Folick et al., 2015). It is tempting to speculate that a related pathway involving lipases and lipid chaperones may activate NHR-49 via a different lipid ligand to achieve longevity in the *glp-1* and other contexts.

#### 4.2.2 Role of NHR-49 in longevity-promoting dietary restriction

Dietary restriction (DR) is an evolutionarily conserved method that achieves substantial lifespan extension in many species. In *C. elegans*, DR can be accomplished by complete food removal (starvation), partial food removal, use of bacteria with a compromised nutrient content, or use of mutants such as *eat-2*, which reduces pharyngeal pumping and hence reduces food intake (Walker et al., 2005; Kapahi et al., 2017). Perhaps in line with the different ways of achieving DR, the requirements for individual genes in these paradigms is not uniform (Walker et al., 2005). *nhr-49* is essential to achieve longevity in several dietary and genetic models of DR (Figure 2). *nhr-49* mutation strongly attenuates lifespan extension in a DR model of total food deprivation, reflecting starvation (Marcellino et al., 2018). Similarly, DR-induced by *Escherichia coli* mutants with intracellular glucose depletion activates a pro-longevity pathway that involves NHR-49, MDT-15, and a specific isoform of the pro-longevity kinase AMPK (Jeong et al., 2023). Requirements for *nhr-49* also extend to genetic models of DR. Specifically, depletion of *mekk-3/Mitogen-activated protein kinase kinase 3* promotes a DR-like state that extends lifespan without affecting food intake, and longevity in this model requires *nhr-49* (Chamoli et al., 2014). The same study also found that *nhr-49* RNAi significantly shortened the longevity of the *eat-2* mutant (Chamoli et al., 2014), while another study observed residual longevity in a similar experiment (Heestand et al., 2013).

#### 4.2.3 *nhr-49* is required for the longevity of some but not all mitochondrial mutants

Mitochondrial function is another process that plays an extensive role in the regulation of *C. elegans* longevity; like DR, this appears to be a conserved mechanism by which eukaryotes can achieve longevity. In *C. elegans*, mutation of some mitochondrial genes causes a dramatic lifespan extension. In particular, mutations in different mitochondrial ETC complex genes yield substantive lifespan extension. Disrupting complexes I-V affects metabolism, causing the worm to enter a starvation-like state (Zuryn et al., 2010). Notably, *nhr-49* is required for the increased longevity conferred by the mitochondrial complex III subunit gene *isp-1* partial loss-of-function allele, *qm150* (Khan et al., 2013). Furthermore, the mitochondrial iron-sulfur cluster assembly protein (ISCU-1) suppresses SKN-1 and NHR-49 through p38 map kinase family member PMK-1 and mitochondrial serine/threonine protein phosphatase 5 PGAM-5 to regulate lifespan as well as oxidative and iron stress response (Sheng et al., 2021). In contrast to these findings, *nhr-49* mutation failed to affect longevity achieved by mutations in ETC complex I, III, and IV genes (Zuryn et al., 2010). In other contexts of disturbed mitochondrial function, *nhr-49* is also dispensable for lifespan extension, such as in worms carrying a mutation in the mitochondrial gene cytochrome C oxidase *cco-1* (also known as *cox-5B*; (Bennett et al., 2017)). Similarly, *nhr-49* is dispensable for the paradoxical effect of the mitochondrial prohibitin complex, whose loss shortens lifespan in wild type but extends it in long-lived mitochondrial mutants (Artal-Sanz and Tavernarakis, 2009). In sum, the requirements for *nhr-49* in mitochondrial longevity are not uniform but rather depend on the specific gene whose mutation promotes longevity.

#### 4.2.4 NHR-49 is required in AMPK/CREB longevity

As noted above, AMPK is a critical effector of DR-induced longevity. AMPK targets and requires the Cyclic AMP-responsive element binding protein (CREB) regulated transcriptional coactivator (CRTC-1) for longevity (Weir et al., 2017). *nhr-49* is required for the AMPK/CRTC circuit to promote longevity (Burkewitz et al., 2015), therefore providing a link between DR, AMPK, CRTC-1, and NHR-49 (Figure 2). Another kinase that is linked to AMPK is Cyclic AMP-dependent Protein Kinase A (PKA), which is upregulated in response to starvation and plays a pro-longevity role though its involvement in lipolysis (Schmeisser et al., 2019). Together with NHR-80, NHR-49 functions in neurons to prolong lifespan upon activation by PKA signaling from muscle tissue (Schmeisser et al., 2019). Therefore, in this context, NHR-49 acts cell-non-autonomously.

#### 4.2.5 NHR-49 promotes low temperature-induced longevity

**FIGURE 3:**
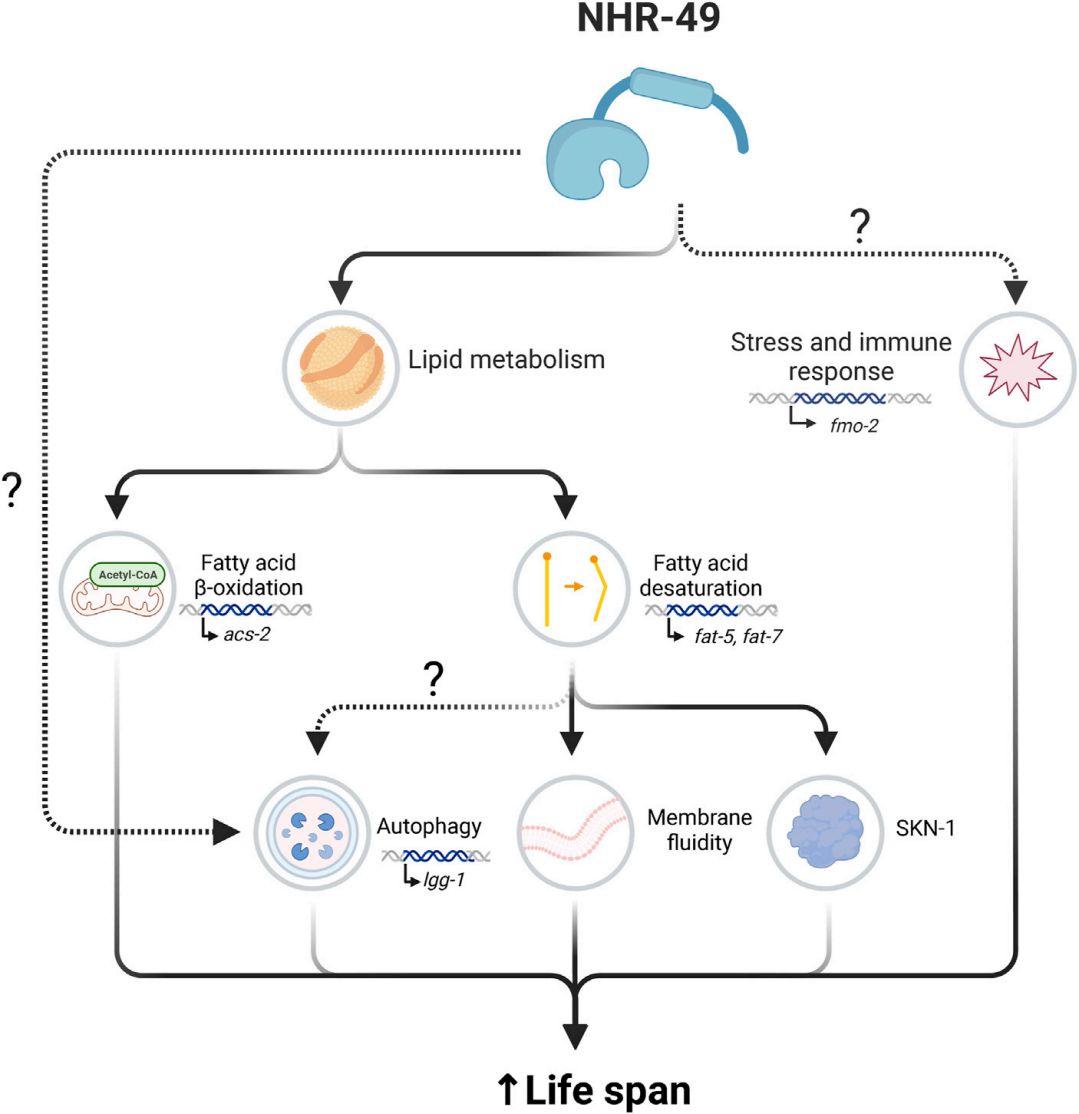
Overview of processes through which NHR-49 promotes longevity. NHR-49 upregulates genes involved in lipid metabolism and stress response regulation to extend lifespan. To promote stress resistance and enhanced immune response, NHR-49 upregulates genes including *fmo-2*. NHR-49’s roles in lipid metabolism include the regulation of fatty acid β-oxidation, wherein NHR-49 upregulates *acs-2*, and fatty acid desaturation, which NHR-49 influences through regulation of *fat-5* and *fat-7*, potentially influencing several linked processes. Collectively, these downstream outputs promote lifespan extension, although not all processes are equally essential in all longevity contexts. Created with BioRender.com.

In the wild, *C. elegans* must adapt to varying temperatures. In the laboratory, worms are usually cultured at 15°C–16°C, 20°C, or 25°C, and lifespan is inversely correlated with temperature (Klass, 1977). *nhr-49* mutation does not affect lifespan at 25°C, but is required for the long lifespan of animals grown at 15°C (Lee et al., 2019). At this temperature, NHR-49 cooperates with MDT-15 to promote maintenance of a proper saturated/unsaturated fatty acid ratio (Lee et al., 2019), promoting lipid homeostasis at lower temperatures (Figures 2, 3). At 15°C, membranes maintain fluidity by increasing the proportion of unsaturated phospholipids. PAQR-2 and its partner IGLR-2 promote adaptation to cold stress by modulating fatty acid desaturation (Svensk et al., 2016). Loss of *paqr-2* causes worm lethality at 15°C because these animals have higher levels of saturated fatty acids, which causes membrane rigidity and thus dysfunction (Svensson et al., 2011; Svensk et al., 2013; Svensk et al., 2016). These phenotypes can be rescued by gain-of-function mutations in *nhr-49* or *mdt-1*5, or by overexpression of SBP-1 (the *C. elegans* ortholog of sterol regulatory element binding proteins (SREBPs)), all of which upregulate fatty acid desaturase genes such as *fat-6* and *fat-7* (Svensk et al., 2013). Indeed, the *fat-6;fat-7* double mutant showed similar cold sensitivity to *paqr-2* mutants at 15°C (Brock et al., 2007). This suggests that NHR-49 may be activated downstream of PAQR-2 to increase phospholipid desaturation and thus membrane fluidity.

Interestingly, although the *paqr-2;nhr-49* double mutant is synthetic lethal, the *paqr-2;nhr-80* double mutant is not. Additionally, loss of *nhr-80* in the *paqr-2* background can rescue lethality at 15°C, suggesting opposing functions for *nhr-49* and *nhr-80* in these conditions (Svensson et al., 2011), despite the fact that both *nhr-49* and *nhr-80* are required to express fatty acid desaturases such as *fat-6* and *fat-7*; perhaps this difference is due to the fact that *nhr-49*, but not *nhr-80*, is required to express *fat-5* (Van Gilst et al., 2005a; Goudeau et al., 2011). Thus, although NHR-80 and NHR-49 are thought to dimerize to regulate fatty acid desaturation (Pathare et al., 2012), they may also have non-overlapping functions.

Besides affecting lipid homeostasis, NHR-49 and MDT-15 also promote proteostasis at 15°C (Lee et al., 2019). This temperature prolongs lifespan in part by reducing protein aggregation due to a lower amount of partially unfolded monomer states that may aggregate (Rosa et al., 2017). Cold-induced protein aggregation can be resolved through induction of the cold sensor channel TRPA-1 that results in upregulation of trypsin-like activity of *C. elegans* proteasome activator subunit PSME-3 (Lee et al., 2023). TRPA-1 signals to PSME-3 through NHR-49, further implicating NHR-49 in regulating cold-induced longevity via modulation of proteostasis (Lee et al., 2023). NHR-49 therefore appears to operate in multiple pathways and affect multiple processes to promote longevity at lower temperatures.

#### 4.2.6 Long-lived contexts/pathways where *nhr-49* is dispensable

Although *nhr-49* is essential for longevity in many situations, it is clearly dispensable in others. Variable requirements for *nhr-49* in long-lived worms with reduced mitochondrial activity are noted above, and other examples exist. Most prominently, insulin/insulin-like growth factor signaling (IIS) is a conserved signaling pathway whose reduced activity substantially extends lifespan in many species, including *C. elegans* (Friedman and Johnson, 1988; Kenyon et al., 1993). However, the *nhr-49(nr2041)* mutation had no impact on the long lifespans of the insulin receptor mutant alleles *daf-2(e1368)* and *daf-2(e1370)* (Ratnappan et al., 2014). Therefore, although NHR-49 regulates lifespan in *C. elegans*, it is just one of several effectors in longevity-promoting pathways.

### 4.3 Mechanisms of NHR-49 lifespan regulation

The previous section describes the role of *nhr-49* in the regulation in longevity, but what biological processes and pathways does it regulate that extend lifespan? Below we summarize the current level of understanding and highlight some future areas of research.

As noted, NHR-49 was originally identified as a regulator of lipid metabolism (Van Gilst et al., 2005a; Van Gilst et al., 2005b). Given the important role that lipid metabolism plays in health and lifespan it was therefore logical to consider that NHR-49’s contribution to longevity would entail modulation of these processes, and this indeed is an important contribution of NHR-49. *nhr-49* is required to express genes involved in fatty acid β-oxidation and in fatty acid desaturation, and both lipid storage and saturation levels are important contributors to lifespan extension (Van Gilst et al., 2005a; Taubert et al., 2006; O’Rourke and Ruvkun, 2013; Ratnappan et al., 2014; Han et al., 2017) (Figure 3).

#### 4.3.1 NHR-49 regulates lifespan through fatty acid desaturation

Fatty acid desaturation is important for the normal lifespan of wild-type worms and to achieve the extended lifespan of several long-lived mutants, and certain unsaturated fatty acids are sufficient to extend the lifespan of wild-type worms (Wang et al., 2008; O’Rourke and Ruvkun, 2013; Han et al., 2017). NHR-49 plays a vital role in this regulation, as NHR-49 mediated production of unsaturated fatty acids is involved in several longevity contexts. NHR-49 driven activation of the fatty acid desaturase genes *fat-5*, *-6*, and *-7* occurs in worms growing at 16°C and also contributes to the extended lifespan of animals experiencing glucose restriction (Ratnappan et al., 2014; Lee et al., 2019; Jeong et al., 2023). However, perhaps the best-characterized context is the *glp-1* mutant, which upregulates *fat-5*, *-6*, and *fat-7* (Goudeau et al., 2011; Ratnappan et al., 2014). The key transcription factors driving these gene activations are NHR-49 and its binding partner NHR-80 (Arda et al., 2010; Pathare et al., 2012). It is therefore possible that these two NHRs form a dimer that activate pro-longevity genes in the *glp-1* mutant. Interestingly, the short lifespan of *glp-1;nhr-80* double mutant is completely rescued by dietary addition of oleic acid, the product of the FAT-6 and FAT-7 enzymes (Goudeau et al., 2011); in contrast, oleic acid supplementation only partially rescues the short lifespan of *glp-1;nhr-49* mutants (Ratnappan et al., 2014). Thus, this implicates NHR-49 in the regulation of processes other than fatty acid desaturation (see below), and suggests that, although an NHR-80–NHR-49 dimer may promote fatty acid desaturation, other pro-longevity NHR-49 dimers may also be required.

How does *nhr-49*-dependent modulation of unsaturated fatty acid content affect *C. elegans* lifespan? First, the SKN-1 pathway is also upregulated in the *glp-1* mutant (Steinbaugh et al., 2015). The desaturases *fat-6* and *-7*, oleic acid, and the lysosomal lipases *lipl-1* and *-3* promote SKN-1 nuclear translocation, where it upregulates numerous genes, including fatty acid β-oxidation and desaturation genes (Steinbaugh et al., 2015). NHR-49–NHR-80 and SKN-1 signaling may therefore undergo positive feedback regulation to reinforce pro-longevity gene programs.

Second, *nhr-49*-dependent increase in unsaturated fatty acid content affects autophagy, which is transcriptionally induced in and required for lifespan extension in several long-lived contexts (Jia and Levine, 2007; Hansen et al., 2008; Tóth et al., 2008; O’Rourke and Ruvkun, 2013; Nieto-Torres and Hansen, 2021). This is evident in worms wherein yolk proteins are depleted, which enhances autophagy and lysosomal lipolysis and extends longevity (Seah et al., 2016). In these worms, *nhr-49* is required for autophagy activation and lifespan extension. Autophagy is also induced by the ω-6 PUFAs γ-linolenic acid and arachidonic acid, which are produced by FAT-7 (Chen et al., 2019). Such upregulation of autophagy occurs at low temperatures (15°C) when the adiponectin receptor PAQR-2 activates NHR-49 (Chen et al., 2019). Interestingly, in animals experiencing hypoxia, *nhr-49* is also required for autophagy, although due to a requirement in the induction of autophagy genes, not fatty acid desaturase genes (Doering et al., 2022). Whether autophagy genes are also regulated by NHR-49 in longevity is not yet known. Overall, this suggests that NHR-49 extends lifespan partially through its effects on autophagy, perhaps both directly and via fatty acid desaturation.

Third, fatty acid desaturation adapts cell and organelle membrane properties. In stresses such as temperature fluctuations, *C. elegans* regulates fatty acid desaturation to adjust membrane rigidity and fluidity (Zhou et al., 2021). Failure to do so can reduce membrane integrity and compromise organelle function. Glucose restriction extends lifespan and promotes membrane fluidity via an AMPK–neuropeptide–PAQR-2–NHR-49 signaling pathway that activates fatty acid desaturase genes, resulting in increased desaturation of membrane lipid fatty acyl tails (Jeong et al., 2023). NHR-49 functions cell non-autonomously in this context, with neuronal or intestinal expression sufficient to rescue glucose restriction-induced longevity in *nhr-49* mutants. In sum, multiple signaling and structural processes are likely affected by *nhr-49*-dependent changes in fatty acids desaturation to extend *C. elegans* lifespan.

#### 4.3.2 NHR-49 regulates lifespan through fatty acid β-oxidation

NHR-49 also regulates fatty acid β-oxidation, which breaks down fatty acids to produce acetyl-CoA. This role is also important for longevity. Specifically, *nhr-49* is required to induce several fatty acid β-oxidation genes in long-lived *glp-1* mutants (Ratnappan et al., 2014). These animals lack a germline, and NHR-49 driven catabolism of fatty acids is a mechanism of eliminating excess fat that is normally allocated for reproduction, thus restoring metabolic homeostasis. Accordingly, the depletion of fatty acid β-oxidation genes such as ACSs and CPTs shortened the lifespan of *glp-1* animals, pinpointing this process as important for longevity. In contrast, fatty acid β-oxidation may be dispensable for lifespan extension by glucose restriction (Jeong et al., 2023). In these animals, wherein *nhr-49* and fatty acid desaturase genes are vital for longevity, depletion of *cpt-1* did not shorten lifespan, although other β-oxidation genes were not tested. *nhr-49* driven β-oxidation therefore contributes to lifespan extension in long-lived mutants, although perhaps less broadly than fatty acid desaturation.

*acs-2* is also regulated by NHR-49 in the AMPK/CRTC longevity pathway (Burkewitz et al., 2015). However, it is unclear whether the key NHR-49 activity in this context is fatty acid β-oxidation, and if *acs-2* contributes to longevity. Interestingly, one key output of the AMPK/CRTC pathway is the modulation of mitochondrial structure and function, which NHR-49 may influence both indirectly via its impact on fatty acid catabolism, and directly, as NHR-49 cooperates with NHR-66 to affect mitochondrial morphology (Pathare et al., 2012).

#### 4.3.3 NHR-49 may regulate longevity through stress response regulation

The role of NHR-49 in altering lipid metabolism to promote longevity is well established; however, NHR-49 may also regulate longevity through other mechanisms. In particular, the emerging role of NHR-49 in stress response regulation may be relevant, as stress and innate immune responses are activated in and contribute to many longevity contexts (Figure 1; Figure 3).

As reviewed, stresses such as starvation, oxidative stress, hypoxia, and pathogen infection can cause upregulation of genes via NHR-49. For example, DR-induced lifespan extension requires *nhr-49* and involves upregulation of detoxification genes, several of which are *nhr-49*-dependent (Chamoli et al., 2014). NHR-49 can function together with SKN-1 to cross-regulate responses to oxidative stress resistance (Hu et al., 2018; Frankino et al., 2022), which could also promote longevity (Steinbaugh et al., 2015). Furthermore, loss of *nhr-49* or *mdt-15* increases susceptibility to bacterial and fungal infections, decreasing *C. elegans* survival on pathogens (Hummell et al., 2021; Naim et al., 2021). Some data, however, suggest that the role of NHR-49 in immunity can be uncoupled from its role in longevity, as gene expression and tissue specific rescue of NHR-49 yield different outcomes in innate immunity and longevity (Naim et al., 2021). Finally, NHR-49 also upregulates *fmo-2*, which regulates life and healthspan in different stress contexts such as hypoxia, dietary restriction, and oxidative stress (Leiser et al., 2015; Huang et al., 2021; Wani et al., 2021). Therefore, NHR-49 may promote longevity in *C. elegans* partially through its role in stress response.

## 5 Challenges and future directions

NHR-49 has emerged as a key effector in stress response and pro-longevity contexts, but many questions about its function and regulation remain. For example, although several *nhr-49*-dependent genes and processes have been identified, it remains unknown whether any of these genes are regulated directly by NHR-49. Approaches such as chromatin immunoprecipitation have provided insight into direct targets of other pro-longevity transcription factors such as DAF-16, as well as its regulatory relationship with other transcription factors (Oh et al., 2006; Kumar et al., 2015; Lin et al., 2018). The fact that no such datasets are yet available suggest that detecting NHR-49’s genomic locations is challenging. The reliance of the above methods on antibodies, of which to our knowledge none are available for NHR-49, represents a challenge, albeit one that can perhaps be solved by the use of genome-edited, tagged NHR-49; alternative methods such as DNA adenine methyltransferase identification are also an option (Schuster et al., 2010). However, low expression of NHR-49 and/or divergent genome occupancy in different tissues may pose additional challenges. *In vitro* approaches to identify NHR-49 binding sites could represent a suitable avenue, as done for DAF-12 (Shostak et al., 2004), but would require *in vivo* validation. The possibility that NHR-49 may occupy different genomic sites in different tissues represents another challenge, and highlights that the genes regulated by NHR-49 in each tissue are not known. As tissue specific transcriptome analysis has become more common (Kaletsky et al., 2018), including in longitudinal settings (Wang et al., 2022), it would be exciting to learn about NHR-49’s tissue specific gene regulatory activities, especially as some tissues such as neurons appear particularly important for *nhr-49*-dependent longevity.

The above questions revolve around NHR-49’s outputs, which, despite some knowledge gaps, are relatively well understood. Less is known about how NHR-49 itself is regulated. In particular, it remains unclear whether or not NHR-49 is controlled by a ligand, as might be expected for an NHR. Evidence in support of such regulation exists. First, several fatty acids and derivative molecules evoke molecular (gene expression) or phenotypic (lifespan extension) effects that depend on *nhr-49* (Ma et al., 2015; Qi et al., 2017), suggesting that these molecules act through NHR-49. Furthermore, molecules of the fibrate class, synthetic ligands of mammalian PPARα (Montaigne et al., 2021), also induce *nhr-49*-dependent lifespan extension in *C. elegans* (Brandstädt et al., 2013). Similarly, the flavonol isorhamnetin reduces overall fat storage in a manner that depends on *nhr-49* (Farias-Pereira et al., 2020). Finally, NHR-80, which binds to and cooperates with NHR-49 to promote lifespan extension, has a *bona fide* ligand in oleylethanolamide (Folick et al., 2015), which binds PPARα (Fu et al., 2003). It is therefore tempting to speculate that NHR-49 may bind a fatty acid or fatty acid derivative in a similar manner to achieve biological outcomes. However, biochemical evidence for an NHR-49 ligand is lacking. Possibly, NHR-49 may act as a “silent partner” to another NHR who is the recipient of a signaling pathway culminating in a ligand, such as NHR-80; it would be interesting to determine whether oleylethanolamide binding increases dimerization of NHR-80 with NHR-49.

Other modes of NHR modulation exist. NHRs are subject to posttranslational modifications, and NHR-49 is phosphorylated at several residues *in vivo* (Gnad et al., 2011). Although it is not yet clear what the impact of these modifications on the NHR-49 activity is, some may contribute to *nhr-49*-dependent stress response and lifespan regulation. Notably, the kinase *hpk-1* is required for the modest increase in NHR-49 protein levels observed in hypoxia, suggesting that this kinase may influence NHR-49 levels (Doering et al., 2022). Combination of proteomics approaches with CRISPR-Cas9 editing of potential sites for posttranslational modification could provide insight into the role of NHR-49’s posttranslational modifications.

Finally, much remains to be learned about how NHR-49 achieves regulatory specificity on its target genes, gene sets, and biological processes. An intriguing possibility is that NHR-49 dimers promote selectivity. Evidence suggests that NHR-66 cooperates with NHR-49 to control the expression of sphingolipid metabolism genes, that NHR-13 and -80 control the expression of fatty acid desaturase genes, and that NHR-79 cooperates with NHR-49 to control genes required for peroxisomal proliferation (Pathare et al., 2012; Zeng et al., 2021). Numerous additional putative NHR-49 dimerization partners have been identified (Li et al., 2004; Taubert et al., 2006; Simonis et al., 2009; Reece-Hoyes et al., 2013; Ratnappan et al., 2016), but their roles in the control of metabolism, stress responses, and lifespan remain unclear. *In vivo* experiments using double mutant analysis should be insightful in revealing regulatory relationships.

The collective work of many labs has revealed complex roles of NHR-49 in the regulation of metabolism, lifespan, and stress responses. The next few years may reveal how NHR-49 achieves these effects, providing exciting new insights into the workings of this important regulator.

## References

[B1] Adeva-Andany M. M. Carneiro-Freire N. Seco-Filgueira M. Fernández-Fernández C. Mouriño-Bayolo D. (2019). Mitochondrial β-oxidation of saturated fatty acids in humans. Mitochondrion 46, 73–90. 10.1016/j.mito.2018.02.009 29551309

[B2] Alam J. Stewart D. Touchard C. Boinapally S. Choi A. M. Cook J. L. (1999). Nrf2, a Cap’n’Collar transcription factor, regulates induction of the heme oxygenase-1 gene. J. Biol. Chem. 274, 26071–26078. 10.1074/jbc.274.37.26071 10473555

[B3] Al-Shehri S. S. (2021). Reactive oxygen and nitrogen species and innate immune response. Biochimie 181, 52–64. 10.1016/j.biochi.2020.11.022 33278558

[B4] An J. H. Blackwell T. K. (2003). SKN-1 links *C. elegans* mesendodermal specification to a conserved oxidative stress response. Genes. Dev. 17, 1882–1893. 10.1101/gad.1107803 12869585 PMC196237

[B5] An J. H. Vranas K. Lucke M. Inoue H. Hisamoto N. Matsumoto K. (2005). Regulation of the *Caenorhabditis elegans* oxidative stress defense protein SKN-1 by glycogen synthase kinase-3. Proc. Natl. Acad. Sci. U. S. A. 102, 16275–16280. 10.1073/pnas.0508105102 16251270 PMC1283458

[B6] Angelo G. Van Gilst M. R. (2009). Starvation protects germline stem cells and extends reproductive longevity in *C. elegans* . Science 326, 954–958. 10.1126/science.1178343 19713489

[B7] Arao Y. Korach K. S. (2021). The physiological role of estrogen receptor functional domains. Essays Biochem. 65, 867–875. 10.1042/EBC20200167 34028522 PMC8611119

[B8] Arda H. E. Taubert S. MacNeil L. T. Conine C. C. Tsuda B. Van Gilst M. (2010). Functional modularity of nuclear hormone receptors in a *Caenorhabditis elegans* metabolic gene regulatory network. Mol. Syst. Biol. 6, 367. 10.1038/msb.2010.23 20461074 PMC2890327

[B9] Artal-Sanz M. Tavernarakis N. (2009). Prohibitin couples diapause signalling to mitochondrial metabolism during ageing in *C. elegans* . Nature 461, 793–797. 10.1038/nature08466 19812672

[B10] Austin J. Kimble J. (1987). glp-1 is required in the germ line for regulation of the decision between mitosis and meiosis in *C. elegans* . Cell. 51, 589–599. 10.1016/0092-8674(87)90128-0 3677168

[B11] Baugh L. R. Hu P. J. (2020). Starvation responses throughout the caenorhabditiselegans life cycle. Genetics 216, 837–878. 10.1534/genetics.120.303565 33268389 PMC7768255

[B12] Bennett C. F. Kwon J. J. Chen C. Russell J. Acosta K. Burnaevskiy N. (2017). Transaldolase inhibition impairs mitochondrial respiration and induces a starvation-like longevity response in *Caenorhabditis elegans* . PLoS Genet. 13, e1006695. 10.1371/journal.pgen.1006695 28355222 PMC5389855

[B13] Berber S. Llamosas E. Thaivalappil P. Boag P. R. Crossley M. Nicholas H. R. (2013). Homeodomain interacting protein kinase (HPK-1) is required in the soma for robust germline proliferation in *C. elegans*: HPK-1 promotes germline proliferation. Dev. Dyn. 242, 1250–1261. 10.1002/dvdy.24023 23904186

[B14] Berber S. Wood M. Llamosas E. Thaivalappil P. Lee K. Liao B. M. (2016). Homeodomain-Interacting Protein Kinase (HPK-1) regulates stress responses and ageing in *C. elegans* . Sci. Rep. 6, 19582. 10.1038/srep19582 26791749 PMC4726358

[B15] Berman J. R. Kenyon C. (2006). Germ-cell loss extends *C. elegans* life span through regulation of DAF-16 by kri-1 and lipophilic-hormone signaling. Cell. 124, 1055–1068. 10.1016/j.cell.2006.01.039 16530050

[B16] Berrabah W. Aumercier P. Lefebvre P. Staels B. (2011). Control of nuclear receptor activities in metabolism by post-translational modifications. FEBS Lett. 585, 1640–1650. 10.1016/j.febslet.2011.03.066 21486568

[B17] Blackwell T. K. Steinbaugh M. J. Hourihan J. M. Ewald C. Y. Isik M. (2015). SKN-1/Nrf, stress responses, and aging in *Caenorhabditis elegans* . Free Radic. Biol. Med. 88, 290–301. 10.1016/j.freeradbiomed.2015.06.008 26232625 PMC4809198

[B18] Brandstädt S. Schmeisser K. Zarse K. Ristow M. (2013). Lipid-lowering fibrates extend *C. elegans* lifespan in a NHR-49/PPARalpha-dependent manner. Aging (Albany NY) 5, 270–275. 10.18632/aging.100548 23603800 PMC3651519

[B19] Brelivet Y. Kammerer S. Rochel N. Poch O. Moras D. (2004). Signature of the oligomeric behaviour of nuclear receptors at the sequence and structural level. EMBO Rep. 5, 423–429. 10.1038/sj.embor.7400119 15105832 PMC1299030

[B20] Brock T. J. Browse J. Watts J. L. (2006). Genetic regulation of unsaturated fatty acid composition in *C. elegans* . PLoS Genet. 2, e108. 10.1371/journal.pgen.0020108 16839188 PMC1500810

[B21] Brock T. J. Browse J. Watts J. L. (2007). Fatty acid desaturation and the regulation of adiposity in *Caenorhabditis elegans* . Genetics 176, 865–875. 10.1534/genetics.107.071860 17435249 PMC1894614

[B22] Brunquell J. Morris S. Lu Y. Cheng F. Westerheide S. D. (2016). The genome-wide role of HSF-1 in the regulation of gene expression in *Caenorhabditis elegans* . BMC Genomics 17, 559. 10.1186/s12864-016-2837-5 27496166 PMC4975890

[B23] Bulcha J. T. Giese G. E. Ali M. Z. Lee Y.-U. Walker M. D. Holdorf A. D. (2019). A persistence detector for metabolic network rewiring in an animal. Cell. Rep. 26, 460–468. 10.1016/j.celrep.2018.12.064 30625328 PMC6368391

[B24] Burkewitz K. Morantte I. Weir H. J. M. Yeo R. Zhang Y. Huynh F. K. (2015). Neuronal CRTC-1 governs systemic mitochondrial metabolism and lifespan via a catecholamine signal. Cell. 160, 842–855. 10.1016/j.cell.2015.02.004 25723162 PMC4392909

[B25] Carreau A. El Hafny-Rahbi B. Matejuk A. Grillon C. Kieda C. (2011). Why is the partial oxygen pressure of human tissues a crucial parameter? Small molecules and hypoxia. J. Cell. Mol. Med. 15, 1239–1253. 10.1111/j.1582-4934.2011.01258.x 21251211 PMC4373326

[B26] Chamoli M. Singh A. Malik Y. Mukhopadhyay A. (2014). A novel kinase regulates dietary restriction-mediated longevity in *Caenorhabditis elegans* . Aging Cell. 13, 641–655. 10.1111/acel.12218 24655420 PMC4326946

[B27] Chapin H. C. Okada M. Merz A. J. Miller D. L. (2015). Tissue-specific autophagy responses to aging and stress in *C. elegans* . Aging (Albany NY) 7, 419–434. 10.18632/aging.100765 26142908 PMC4505168

[B28] Chen Y.-L. Tao J. Zhao P.-J. Tang W. Xu J.-P. Zhang K.-Q. (2019). Adiponectin receptor PAQR-2 signaling senses low temperature to promote *C. elegans* longevity by regulating autophagy. Nat. Commun. 10, 2602. 10.1038/s41467-019-10475-8 31197136 PMC6565724

[B29] Choudhry H. Harris A. L. (2018). Advances in hypoxia-inducible factor biology. Cell. Metab. 27, 281–298. 10.1016/j.cmet.2017.10.005 29129785

[B30] Das R. Melo J. A. Thondamal M. Morton E. A. Cornwell A. B. Crick B. (2017). The homeodomain-interacting protein kinase HPK-1 preserves protein homeostasis and longevity through master regulatory control of the HSF-1 chaperone network and TORC1-restricted autophagy in *Caenorhabditis elegans* . PLoS Genet. 13, e1007038. 10.1371/journal.pgen.1007038 29036198 PMC5658188

[B31] Dasgupta M. Shashikanth M. Gupta A. Sandhu A. De A. Javed S. (2020). NHR-49 transcription factor regulates immunometabolic response and survival of *Caenorhabditis elegans* during *Enterococcus faecalis* infection. Infect. Immun. 88, e00130-20. 10.1128/IAI.00130-20 32482643 PMC7375755

[B32] Doering K. R. S. Cheng X. Milburn L. Ratnappan R. Ghazi A. Miller D. L. (2022). Nuclear Hormone Receptor NHR-49 acts in parallel with HIF-1 to promote hypoxia adaptation in *Caenorhabditis elegans* . Elife 11, e67911. 10.7554/eLife.67911 35285794 PMC8959602

[B33] Epstein A. C. Gleadle J. M. McNeill L. A. Hewitson K. S. O’Rourke J. Mole D. R. (2001). *C. elegans* EGL-9 and mammalian homologs define a family of dioxygenases that regulate HIF by prolyl hydroxylation. Cell. 107, 43–54. 10.1016/s0092-8674(01)00507-4 11595184

[B34] Ermolaeva M. A. Schumacher B. (2014). Insights from the worm: the *C. elegans* model for innate immunity. Semin. Immunol. 26, 303–309. 10.1016/j.smim.2014.04.005 24856329 PMC4248339

[B35] Eustice M. Konzman D. Reece J. M. Ghosh S. Alston J. Hansen T. (2022). Nutrient sensing pathways regulating adult reproductive diapause in *C. elegans* . PLoS One 17, e0274076. 10.1371/journal.pone.0274076 36112613 PMC9480990

[B36] Farias-Pereira R. Savarese J. Yue Y. Lee S.-H. Park Y. (2020). Fat-lowering effects of isorhamnetin are via NHR-49-dependent pathway in *Caenorhabditis elegans* . Curr. Res. Food Sci. 2, 70–76. 10.1016/j.crfs.2019.11.002 32914113 PMC7473354

[B37] Folick A. Oakley H. D. Yu Y. Armstrong E. H. Kumari M. Sanor L. (2015). Aging. Lysosomal signaling molecules regulate longevity in *Caenorhabditis elegans* . Science 347, 83–86. 10.1126/science.1258857 25554789 PMC4425353

[B38] Frankino P. A. Siddiqi T. F. Bolas T. Bar-Ziv R. Gildea H. K. Zhang H. (2022). SKN-1 regulates stress resistance downstream of amino catabolism pathways. iScience 25, 104571. 10.1016/j.isci.2022.104571 35784796 PMC9240870

[B39] Friedman D. B. Johnson T. E. (1988). A mutation in the age-1 gene in *Caenorhabditis elegans* lengthens life and reduces hermaphrodite fertility. Genetics 118, 75–86. 10.1093/genetics/118.1.75 8608934 PMC1203268

[B40] Frigo D. E. Bondesson M. Williams C. (2021). Nuclear receptors: from molecular mechanisms to therapeutics. Essays Biochem. 65, 847–856. 10.1042/EBC20210020 PMC8628184 34825698

[B41] Fu J. Gaetani S. Oveisi F. Lo Verme J. Serrano A. Rodríguez De Fonseca F. (2003). Oleylethanolamide regulates feeding and body weight through activation of the nuclear receptor PPAR-alpha. Nature 425, 90–93. 10.1038/nature01921 12955147

[B42] Fulda S. Gorman A. M. Hori O. Samali A. (2010). Cellular stress responses: cell survival and cell death. Int. J. Cell. Biol. 2010, 214074. 10.1155/2010/214074 20182529 PMC2825543

[B43] Galluzzi L. Yamazaki T. Kroemer G. (2018). Linking cellular stress responses to systemic homeostasis. Nat. Rev. Mol. Cell. Biol. 19, 731–745. 10.1038/s41580-018-0068-0 30305710

[B44] Gammon D. B. (2017). *Caenorhabditis elegans* as an emerging model for virus-host interactions. J. Virol. 91, e00509–e00517. 10.1128/JVI.00509-17 28931683 PMC5686719

[B45] Garsin D. A. Sifri C. D. Mylonakis E. Qin X. Singh K. V. Murray B. E. (2001). A simple model host for identifying Gram-positive virulence factors. Proc. Natl. Acad. Sci. U. S. A. 98, 10892–10897. 10.1073/pnas.191378698 11535834 PMC58570

[B46] Gerisch B. Tharyan R. G. Mak J. Denzel S. I. Popkes-van Oepen T. Henn N. (2020). HLH-30/TFEB is a master regulator of reproductive quiescence. Dev. Cell. 53, 316–329. 10.1016/j.devcel.2020.03.014 32302543

[B47] Giese G. E. Walker M. D. Ponomarova O. Zhang H. Li X. Minevich G. (2020). *Caenorhabditis elegans* methionine/S-adenosylmethionine cycle activity is sensed and adjusted by a nuclear hormone receptor. Elife 9, e60259. 10.7554/eLife.60259 33016879 PMC7561351

[B48] Gnad F. Gunawardena J. Mann M. (2011). Phosida 2011: the posttranslational modification database. Nucleic Acids Res. 39, D253–D260. 10.1093/nar/gkq1159 21081558 PMC3013726

[B49] Goh G. Y. S. Martelli K. L. Parhar K. S. Kwong A. W. L. Wong M. A. Mah A. (2014). The conserved Mediator subunit MDT-15 is required for oxidative stress responses in *Caenorhabditis elegans* . Aging Cell. 13, 70–79. 10.1111/acel.12154 23957350 PMC4326869

[B50] Goh G. Y. S. Winter J. J. Bhanshali F. Doering K. R. S. Lai R. Lee K. (2018). NHR-49/HNF4 integrates regulation of fatty acid metabolism with a protective transcriptional response to oxidative stress and fasting. Aging Cell. 17, e12743. 10.1111/acel.12743 29508513 PMC5946062

[B51] Goh G. Y. S. Beigi A. Yan J. Doering K. R. S. Taubert S. (2023). Mediator subunit MDT-15 promotes expression of propionic acid breakdown genes to prevent embryonic lethality in *Caenorhabditis elegans* . G3 (Bethesda) 13, jkad087. 10.1093/g3journal/jkad087 37075089 PMC10234398

[B52] Goswamy D. Gonzalez X. Labed S. A. Irazoqui J. E. (2023). *C. elegans* orphan nuclear receptor NHR-42 represses innate immunity and promotes lipid loss downstream of HLH-30/TFEB. Front. Immunol. 14, 1094145. 10.3389/fimmu.2023.1094145 36860863 PMC9968933

[B53] Goudeau J. Bellemin S. Toselli-Mollereau E. Shamalnasab M. Chen Y. Aguilaniu H. (2011). Fatty acid desaturation links germ cell loss to longevity through NHR-80/HNF4 in *C. elegans* . PLoS Biol. 9, e1000599. 10.1371/journal.pbio.1000599 21423649 PMC3057950

[B54] Gracida X. Eckmann C. R. (2013). Fertility and germline stem cell maintenance under different diets requires nhr-114/HNF4 in *C. elegans* . Curr. Biol. 23, 607–613. 10.1016/j.cub.2013.02.034 23499532

[B55] Halliwell B. (1991). Reactive oxygen species in living systems: source, biochemistry, and role in human disease. Am. J. Med. 91, 14S–S22. 10.1016/0002-9343(91)90279-7 1928205

[B56] Han S. Schroeder E. A. Silva-García C. G. Hebestreit K. Mair W. B. Brunet A. (2017). Mono-unsaturated fatty acids link H3K4me3 modifiers to *C. elegans* lifespan. Nature 544, 185–190. 10.1038/nature21686 28379943 PMC5391274

[B57] Hansen M. Chandra A. Mitic L. L. Onken B. Driscoll M. Kenyon C. (2008). A role for autophagy in the extension of lifespan by dietary restriction in *C. elegans* . PLoS Genet. 4, e24. 10.1371/journal.pgen.0040024 18282106 PMC2242811

[B58] Harvald E. B. Sprenger R. R. Dall K. B. Ejsing C. S. Nielsen R. Mandrup S. (2017). Multi-omics analyses of starvation responses reveal a central role for lipoprotein metabolism in acute starvation survival in *C. elegans* . Cell. Syst. 5, 38–52. 10.1016/j.cels.2017.06.004 28734827

[B59] Heestand B. N. Shen Y. Liu W. Magner D. B. Storm N. Meharg C. (2013). Dietary restriction induced longevity is mediated by nuclear receptor NHR-62 in *Caenorhabditis elegans* . PLoS Genet. 9, e1003651. 10.1371/journal.pgen.1003651 23935515 PMC3723528

[B60] Hoffmann J. M. Partridge L. (2015). Nuclear hormone receptors: roles of xenobiotic detoxification and sterol homeostasis in healthy aging. Crit. Rev. Biochem. Mol. Biol. 50, 380–392. 10.3109/10409238.2015.1067186 26383043

[B61] Horikawa M. Sakamoto K. (2009). Fatty-acid metabolism is involved in stress-resistance mechanisms of *Caenorhabditis elegans* . Biochem. Biophys. Res. Commun. 390, 1402–1407. 10.1016/j.bbrc.2009.11.006 19896458

[B62] Hu Q. D’Amora D. R. MacNeil L. T. Walhout A. J. M. Kubiseski T. J. (2018). The *Caenorhabditis elegans* oxidative stress response requires the NHR-49 transcription factor. G3 (Bethesda) 8, 3857–3863. 10.1534/g3.118.200727 30297383 PMC6288832

[B63] Hu P. J. (2007). “Dauer,” in WormBook, 1–19. 10.1895/wormbook.1.144.1 PMC2890228 17988074

[B64] Huang W. Li Z. Xu Y. Wang W. Zhou M. Zhang P. (2014). PKG and NHR-49 signalling co-ordinately regulate short-term fasting-induced lysosomal lipid accumulation in *C. elegans* . Biochem. J. 461, 509–520. 10.1042/BJ20140191 24854345

[B65] Huang S. Howington M. B. Dobry C. J. Evans C. R. Leiser S. F. (2021). Flavin-containing monooxygenases are conserved regulators of stress resistance and metabolism. Front. Cell. Dev. Biol. 9, 630188. 10.3389/fcell.2021.630188 33644069 PMC7907451

[B66] Hummell N. A. Revtovich A. V. Kirienko N. V. (2021). Novel immune modulators enhance *Caenorhabditis elegans* resistance to multiple pathogens. mSphere 6 (1), e00950–20. 10.1128/mSphere.00950-20 33408224 PMC7845594

[B67] Irazoqui J. E. Troemel E. R. Feinbaum R. L. Luhachack L. G. Cezairliyan B. O. Ausubel F. M. (2010). Distinct pathogenesis and host responses during infection of *C. elegans* by *P. aeruginosa* and *S. aureus* . PLoS Pathog. 6, e1000982. 10.1371/journal.ppat.1000982 20617181 PMC2895663

[B68] Jeong J.-H. Han J.-S. Jung Y. Lee S.-M. Park S.-H. Park M. (2023). A new AMPK isoform mediates glucose-restriction induced longevity non-cell autonomously by promoting membrane fluidity. Nat. Commun. 14, 288. 10.1038/s41467-023-35952-z 36653384 PMC9849402

[B69] Jia K. Levine B. (2007). Autophagy is required for dietary restriction-mediated life span extension in *C. elegans* . Autophagy 3, 597–599. 10.4161/auto.4989 17912023

[B70] Jiang H. Guo R. Powell-Coffman J. A. (2001). The *Caenorhabditis elegans* hif-1 gene encodes a bHLH-PAS protein that is required for adaptation to hypoxia. Proc. Natl. Acad. Sci. U. S. A. 98, 7916–7921. 10.1073/pnas.141234698 11427734 PMC35443

[B71] Kaletsky R. Yao V. Williams A. Runnels A. M. Tadych A. Zhou S. (2018). Transcriptome analysis of adult *Caenorhabditis elegans* cells reveals tissue-specific gene and isoform expression. PLoS Genet. 14, e1007559. 10.1371/journal.pgen.1007559 30096138 PMC6105014

[B72] Kapahi P. Kaeberlein M. Hansen M. (2017). Dietary restriction and lifespan: lessons from invertebrate models. Ageing Res. Rev. 39, 3–14. 10.1016/j.arr.2016.12.005 28007498 PMC5476520

[B73] Kenyon C. Chang J. Gensch E. Rudner A. Tabtiang R. (1993). A *C. elegans* mutant that lives twice as long as wild type. Nature 366, 461–464. 10.1038/366461a0 8247153

[B74] Khan S. H. Okafor C. D. (2022). Interactions governing transcriptional activity of nuclear receptors. Biochem. Soc. Trans. 50, 1941–1952. 10.1042/BST20220338 36524961

[B75] Khan M. H. Ligon M. Hussey L. R. Hufnal B. Farber R. Munkácsy E. (2013). TAF-4 is required for the life extension of isp-1, clk-1 and tpk-1 Mit mutants. Aging (Albany NY) 5, 741–758. 10.18632/aging.100604 24107417 PMC3838777

[B76] Kim D. H. Ewbank J. J. (2018). Signaling in the innate immune response. WormBook 2018, 1–35. 10.1895/wormbook.1.83.2 PMC6369418 26694508

[B77] Klass M. R. (1977). Aging in the nematode *Caenorhabditis elegans*: major biological and environmental factors influencing life span. Mech. Ageing Dev. 6, 413–429. 10.1016/0047-6374(77)90043-4 926867

[B78] Kostrouchova M. Kostrouch Z. (2015). Nuclear receptors in nematode development: natural experiments made by a phylum. Biochim. Biophys. Acta 1849, 224–237. 10.1016/j.bbagrm.2014.06.016 24984201

[B79] Kotulkar M. Robarts D. Apte U. (2023). HNF4α in hepatocyte health and disease. Semin. Liver Dis. 43, 234–244. 10.1055/a-2097-0660 37216979 PMC10947958

[B80] Kumar N. Jain V. Singh A. Jagtap U. Verma S. Mukhopadhyay A. (2015). Genome-wide endogenous DAF-16/FOXO recruitment dynamics during lowered insulin signalling in *C. elegans* . Oncotarget 6, 41418–41433. 10.18632/oncotarget.6282 26539642 PMC4747164

[B81] Lapierre L. R. De Magalhaes Filho C. D. McQuary P. R. Chu C.-C. Visvikis O. Chang J. T. (2013). The TFEB orthologue HLH-30 regulates autophagy and modulates longevity in *Caenorhabditis elegans* . Nat. Commun. 4, 2267. 10.1038/ncomms3267 23925298 PMC3866206

[B82] Lee K. Goh G. Y. S. Wong M. A. Klassen T. L. Taubert S. (2016). Gain-of-Function alleles in *Caenorhabditis elegans* nuclear hormone receptor nhr-49 are functionally distinct. PloS One 11, e0162708. 10.1371/journal.pone.0162708 27618178 PMC5019492

[B83] Lee D. An S. W. A. Jung Y. Yamaoka Y. Ryu Y. Goh G. Y. S. (2019). MDT-15/MED15 permits longevity at low temperature via enhancing lipidostasis and proteostasis. PLoS Biol. 17, e3000415. 10.1371/journal.pbio.3000415 31408455 PMC6692015

[B84] Lee P. Chandel N. S. Simon M. C. (2020). Cellular adaptation to hypoxia through hypoxia inducible factors and beyond. Nat. Rev. Mol. Cell. Biol. 21, 268–283. 10.1038/s41580-020-0227-y 32144406 PMC7222024

[B85] Lee H. J. Alirzayeva H. Koyuncu S. Rueber A. Noormohammadi A. Vilchez D. (2023). Cold temperature extends longevity and prevents disease-related protein aggregation through PA28γ-induced proteasomes. Nat. Aging 3, 546–566. 10.1038/s43587-023-00383-4 37118550 PMC10191861

[B86] Leiser S. F. Miller H. Rossner R. Fletcher M. Leonard A. Primitivo M. (2015). Cell nonautonomous activation of flavin-containing monooxygenase promotes longevity and health span. Sci. (New York, N.Y.) 350, 1375–1378. 10.1126/science.aac9257 PMC4801033 26586189

[B87] Leiteritz A. Baumanns S. Wenzel U. (2020). Amyloid-beta (Aβ1-42)-induced paralysis in *Caenorhabditis elegans* is reduced through NHR-49/PPARalpha. Neurosci. Lett. 730, 135042. 10.1016/j.neulet.2020.135042 32413539

[B88] Lenaz G. (2001). The mitochondrial production of reactive oxygen species: mechanisms and implications in human pathology. IUBMB Life 52, 159–164. 10.1080/15216540152845957 11798028

[B89] Li S. Armstrong C. M. Bertin N. Ge H. Milstein S. Boxem M. (2004). A map of the interactome network of the metazoan *C. elegans* . Science 303, 540–543. 10.1126/science.1091403 14704431 PMC1698949

[B90] Li Y. Padmanabha D. Gentile L. B. Dumur C. I. Beckstead R. B. Baker K. D. (2013). HIF- and non-HIF-regulated hypoxic responses require the estrogen-related receptor in *Drosophila melanogaster* . PLoS Genet. 9, e1003230. 10.1371/journal.pgen.1003230 23382692 PMC3561118

[B91] Liang B. Ferguson K. Kadyk L. Watts J. L. (2010). The role of nuclear receptor NHR-64 in fat storage regulation in *Caenorhabditis elegans* . PLoS One 5, e9869. 10.1371/journal.pone.0009869 20360843 PMC2845610

[B92] Lin X.-X. Sen I. Janssens G. E. Zhou X. Fonslow B. R. Edgar D. (2018). DAF-16/FOXO and HLH-30/TFEB function as combinatorial transcription factors to promote stress resistance and longevity. Nat. Commun. 9, 4400. 10.1038/s41467-018-06624-0 30353013 PMC6199276

[B93] Ma D. K. Li Z. Lu A. Y. Sun F. Chen S. Rothe M. (2015). Acyl-CoA dehydrogenase drives heat adaptation by sequestering fatty acids. Cell. 161, 1152–1163. 10.1016/j.cell.2015.04.026 25981666 PMC4441829

[B94] Ma Q. (2013). Role of nrf2 in oxidative stress and toxicity. Annu. Rev. Pharmacol. Toxicol. 53, 401–426. 10.1146/annurev-pharmtox-011112-140320 23294312 PMC4680839

[B95] Magner D. B. Antebi A. (2008). *Caenorhabditis elegans* nuclear receptors: insights into life traits. Trends Endocrinol. Metab. 19, 153–160. 10.1016/j.tem.2008.02.005 18406164 PMC2744080

[B96] Magner D. B. Wollam J. Shen Y. Hoppe C. Li D. Latza C. (2013). The NHR-8 nuclear receptor regulates cholesterol and bile acid homeostasis in *C. elegans* . Cell. Metab. 18, 212–224. 10.1016/j.cmet.2013.07.007 23931753 PMC3909615

[B97] Marcellino B. K. Ekasumara N. Mobbs C. V. (2018). Dietary restriction and glycolytic inhibition reduce proteotoxicity and extend lifespan via NHR-49. Curr. Neurobiol. 9, 1–7. 30820135 PMC6390974

[B98] Martindale J. L. Holbrook N. J. (2002). Cellular response to oxidative stress: signaling for suicide and survival. J. Cell. Physiol. 192, 1–15. 10.1002/jcp.10119 12115731

[B99] Martineau C. N. Kirienko N. V. Pujol N. (2021). Innate immunity in *C. elegans* . Curr. Top. Dev. Biol. 144, 309–351. 10.1016/bs.ctdb.2020.12.007 33992157 PMC9175240

[B100] Mazure N. M. Pouysségur J. (2010). Hypoxia-induced autophagy: cell death or cell survival? Curr. Opin. Cell. Biol. 22, 177–180. 10.1016/j.ceb.2009.11.015 20022734

[B101] Miranda-Vizuete A. Veal E. A. (2017). *Caenorhabditis elegans* as a model for understanding ROS function in physiology and disease. Redox Biol. 11, 708–714. 10.1016/j.redox.2016.12.020 28193593 PMC5304259

[B102] Montaigne D. Butruille L. Staels B. (2021). PPAR control of metabolism and cardiovascular functions. Nat. Rev. Cardiol. 18, 809–823. 10.1038/s41569-021-00569-6 34127848

[B103] Mouchiroud L. Eichner L. J. Shaw R. J. Auwerx J. (2014). Transcriptional coregulators: fine-tuning metabolism. Cell. Metab. 20, 26–40. 10.1016/j.cmet.2014.03.027 24794975 PMC4079747

[B104] Naim N. Amrit F. R. G. Ratnappan R. DelBuono N. Loose J. A. Ghazi A. (2021). Cell nonautonomous roles of NHR-49 in promoting longevity and innate immunity. Aging Cell. 20, e13413. 10.1111/acel.13413 34156142 PMC8282243

[B105] Nieto-Torres J. L. Hansen M. (2021). Macroautophagy and aging: the impact of cellular recycling on health and longevity. Mol. Asp. Med. 82, 101020. 10.1016/j.mam.2021.101020 PMC8671213 34507801

[B106] O’Rourke E. J. Ruvkun G. (2013). MXL-3 and HLH-30 transcriptionally link lipolysis and autophagy to nutrient availability. Nat. Cell. Biol. 15, 668–676. 10.1038/ncb2741 23604316 PMC3723461

[B107] Oh S. W. Mukhopadhyay A. Dixit B. L. Raha T. Green M. R. Tissenbaum H. A. (2006). Identification of direct DAF-16 targets controlling longevity, metabolism and diapause by chromatin immunoprecipitation. Nat. Genet. 38, 251–257. 10.1038/ng1723 16380712

[B108] Oliveira R. P. Porter Abate J. Dilks K. Landis J. Ashraf J. Murphy C. T. (2009). Condition-adapted stress and longevity gene regulation by *Caenorhabditis elegans* SKN-1/Nrf. Aging Cell. 8, 524–541. 10.1111/j.1474-9726.2009.00501.x 19575768 PMC2776707

[B109] Oswald M. C. W. Garnham N. Sweeney S. T. Landgraf M. (2018). Regulation of neuronal development and function by ROS. FEBS Lett. 592, 679–691. 10.1002/1873-3468.12972 29323696 PMC5888200

[B110] Padmanabha D. Padilla P. A. You Y.-J. Baker K. D. (2015). A HIF-independent mediator of transcriptional responses to oxygen deprivation in *Caenorhabditis elegans* . Genetics 199, 739–748. 10.1534/genetics.114.173989 25552276 PMC4349068

[B111] Palanker L. Tennessen J. M. Lam G. Thummel C. S. (2009). Drosophila HNF4 regulates lipid mobilization and beta-oxidation. Cell. Metab. 9, 228–239. 10.1016/j.cmet.2009.01.009 19254568 PMC2673486

[B112] Park D. Jones K. L. Lee H. Snutch T. P. Taubert S. Riddle D. L. (2012). Repression of a potassium channel by nuclear hormone receptor and TGF-β signaling modulates insulin signaling in *Caenorhabditis elegans* . PLoS Genet. 8, e1002519. 10.1371/journal.pgen.1002519 22359515 PMC3280960

[B113] Pathare P. P. Lin A. Bornfeldt K. E. Taubert S. Van Gilst M. R. (2012). Coordinate regulation of lipid metabolism by novel nuclear receptor partnerships. PLoS Genet. 8, e1002645. 10.1371/journal.pgen.1002645 22511885 PMC3325191

[B114] Pees B. Yang W. Kloock A. Petersen C. Peters L. Fan L. (2021). Effector and regulator: diverse functions of *C. elegans* C-type lectin-like domain proteins. PLoS Pathog. 17, e1009454. 10.1371/journal.ppat.1009454 33793670 PMC8051790

[B115] Pender C. L. Horvitz H. R. (2018). Hypoxia-inducible factor cell non-autonomously regulates *C. elegans* stress responses and behavior via a nuclear receptor. Elife 7, e36828. 10.7554/eLife.36828 30010540 PMC6078495

[B116] Peterson N. D. Cheesman H. K. Liu P. Anderson S. M. Foster K. J. Chhaya R. (2019). The nuclear hormone receptor NHR-86 controls anti-pathogen responses in *C. elegans* . PLoS Genet. 15, e1007935. 10.1371/journal.pgen.1007935 30668573 PMC6358101

[B117] Peterson N. D. Tse S. Y. Huang Q. J. Wani K. A. Schiffer C. A. Pukkila-Worley R. (2023). Non-canonical pattern recognition of a pathogen-derived metabolite by a nuclear hormone receptor identifies virulent bacteria in *C. elegans* . Immunity 56, 768–782.e9. 10.1016/j.immuni.2023.01.027 36804958 PMC10101930

[B118] Powell J. R. Ausubel F. M. (2008). Models of *Caenorhabditis elegans* infection by bacterial and fungal pathogens. Methods Mol. Biol. 415, 403–427. 10.1007/978-1-59745-570-1_24 18370168

[B119] Powell-Coffman J. A. (2010). Hypoxia signaling and resistance in *C. elegans* . Trends Endocrinol. metabolism TEM 21, 435–440. 10.1016/j.tem.2010.02.006 20335046

[B120] Pursiheimo J.-P. Rantanen K. Heikkinen P. T. Johansen T. Jaakkola P. M. (2009). Hypoxia-activated autophagy accelerates degradation of SQSTM1/p62. Oncogene 28, 334–344. 10.1038/onc.2008.392 18931699

[B121] Qi W. Gutierrez G. E. Gao X. Dixon H. McDonough J. A. Marini A. M. (2017). The ω-3 fatty acid α-linolenic acid extends *Caenorhabditis elegans* lifespan via NHR-49/PPARα and oxidation to oxylipins. Aging Cell. 16, 1125–1135. 10.1111/acel.12651 28772063 PMC5595674

[B122] Qin S. Wang Y. Li L. Liu J. Xiao C. Duan D. (2022). Early-life vitamin B12 orchestrates lipid peroxidation to ensure reproductive success via SBP-1/SREBP1 in *Caenorhabditis elegans* . Cell. Rep. 40, 111381. 10.1016/j.celrep.2022.111381 36130518

[B123] Rackles E. Witting M. Forné I. Zhang X. Zacherl J. Schrott S. (2021). Reduced peroxisomal import triggers peroxisomal retrograde signaling. Cell. Rep. 34, 108653. 10.1016/j.celrep.2020.108653 33472070

[B124] Rajan M. Anderson C. P. Rindler P. M. Romney S. J. Ferreira Dos Santos M. C. Gertz J. (2019). NHR-14 loss of function couples intestinal iron uptake with innate immunity in *C. elegans* through PQM-1 signaling. Elife 8, e44674. 10.7554/eLife.44674 31532389 PMC6777940

[B125] Ratnappan R. Amrit F. R. G. Chen S.-W. Gill H. Holden K. Ward J. (2014). Germline signals deploy NHR-49 to modulate fatty-acid β-oxidation and desaturation in somatic tissues of *C. elegans* . PLoS Genet. 10, e1004829. 10.1371/journal.pgen.1004829 25474470 PMC4256272

[B126] Ratnappan R. Ward J. D. Yamamoto K. R. Ghazi A. (2016). Nuclear hormone receptors as mediators of metabolic adaptability following reproductive perturbations. Worm 5, e1151609. 10.1080/21624054.2016.1151609 27073739 PMC4805359

[B127] Reece-Hoyes J. S. Pons C. Diallo A. Mori A. Shrestha S. Kadreppa S. (2013). Extensive rewiring and complex evolutionary dynamics in a *C. elegans* multiparameter transcription factor network. Mol. Cell. 51, 116–127. 10.1016/j.molcel.2013.05.018 23791784 PMC3794439

[B128] Rinaldo C. Prodosmo A. Siepi F. Soddu S. (2007). HIPK2: a multitalented partner for transcription factors in DNA damage response and development. Biochem. Cell. Biol. = Biochimie Biol. Cell. 85, 411–418. 10.1139/O07-071 17713576

[B129] Rosa M. Roberts C. J. Rodrigues M. A. (2017). Connecting high-temperature and low-temperature protein stability and aggregation. PLoS One 12, e0176748. 10.1371/journal.pone.0176748 28472066 PMC5417562

[B130] Sala A. J. Grant R. A. Imran G. Morton C. Brielmann R. M. Bott L. C. (2023). Nuclear receptor signaling via NHR-49/MDT-15 regulates stress resilience and proteostasis in response to reproductive and metabolic cues. Genetics, 2023.04.25.537803. 10.1101/2023.04.25.537803 PMC11216168 38816072

[B131] Samokhvalov V. Scott B. A. Crowder C. M. (2008). Autophagy protects against hypoxic injury in *C. elegans* . Autophagy 4, 1034–1041. 10.4161/auto.6994 18849662 PMC3670992

[B132] Savini M. Folick A. Lee Y.-T. Jin F. Cuevas A. Tillman M. C. (2022). Lysosome lipid signalling from the periphery to neurons regulates longevity. Nat. Cell. Biol. 24, 906–916. 10.1038/s41556-022-00926-8 35681008 PMC9203275

[B133] Schmeisser S. Li S. Bouchard B. Ruiz M. Des Rosiers C. Roy R. (2019). Muscle-specific lipid hydrolysis prolongs lifespan through global lipidomic remodeling. Cell. Rep. 29, 4540–4552. 10.1016/j.celrep.2019.11.090 31875559

[B134] Scholtes C. Giguère V. (2022). Transcriptional control of energy metabolism by nuclear receptors. Nat. Rev. Mol. Cell. Biol. 23, 750–770. 10.1038/s41580-022-00486-7 35577989

[B135] Schulenburg H. Félix M.-A. (2017). The natural biotic environment of *Caenorhabditis elegans* . Genetics 206, 55–86. 10.1534/genetics.116.195511 28476862 PMC5419493

[B136] Schulenburg H. Hoeppner M. P. Weiner J. Bornberg-Bauer E. (2008). Specificity of the innate immune system and diversity of C-type lectin domain (CTLD) proteins in the nematode *Caenorhabditis elegans* . Immunobiology 213, 237–250. 10.1016/j.imbio.2007.12.004 18406370

[B137] Schuster E. McElwee J. J. Tullet J. M. A. Doonan R. Matthijssens F. Reece-Hoyes J. S. (2010). DamID in *C. elegans* reveals longevity-associated targets of DAF-16/FoxO. Mol. Syst. Biol. 6, 399. 10.1038/msb.2010.54 20706209 PMC2950082

[B138] Seah N. E. de Magalhaes Filho C. D. Petrashen A. P. Henderson H. R. Laguer J. Gonzalez J. (2016). Autophagy-mediated longevity is modulated by lipoprotein biogenesis. Autophagy 12, 261–272. 10.1080/15548627.2015.1127464 26671266 PMC4836030

[B139] Settembre C. De Cegli R. Mansueto G. Saha P. K. Vetrini F. Visvikis O. (2013). TFEB controls cellular lipid metabolism through a starvation-induced autoregulatory loop. Nat. Cell. Biol. 15, 647–658. 10.1038/ncb2718 23604321 PMC3699877

[B140] Sever R. Glass C. K. (2013). Signaling by nuclear receptors. Cold Spring Harb. Perspect. Biol. 5, a016709. 10.1101/cshperspect.a016709 23457262 PMC3578364

[B141] Shen C. Nettleton D. Jiang M. Kim S. K. Powell-Coffman J. A. (2005). Roles of the HIF-1 hypoxia-inducible factor during hypoxia response in *Caenorhabditis elegans* . J. Biol. Chem. 280, 20580–20588. 10.1074/jbc.M501894200 15781453

[B142] Sheng Y. Yang G. Casey K. Curry S. Oliver M. Han S. M. (2021). A novel role of the mitochondrial iron-sulfur cluster assembly protein ISCU-1/ISCU in longevity and stress response. Geroscience 43, 691–707. 10.1007/s11357-021-00327-z 33527323 PMC8110660

[B143] Shields H. J. Traa A. Van Raamsdonk J. M. (2021). Beneficial and detrimental effects of reactive oxygen species on lifespan: a comprehensive review of comparative and experimental studies. Front. Cell. Dev. Biol. 9, 628157. 10.3389/fcell.2021.628157 33644065 PMC7905231

[B144] Shostak Y. Van Gilst M. R. Antebi A. Yamamoto K. R. (2004). Identification of *C. elegans* DAF-12-binding sites, response elements, and target genes. Genes. Dev. 18, 2529–2544. 10.1101/gad.1218504 15489294 PMC529540

[B145] Sies H. Jones D. P. (2020). Reactive oxygen species (ROS) as pleiotropic physiological signalling agents. Nat. Rev. Mol. Cell. Biol. 21, 363–383. 10.1038/s41580-020-0230-3 32231263

[B146] Sies H. Belousov V. V. Chandel N. S. Davies M. J. Jones D. P. Mann G. E. (2022). Defining roles of specific reactive oxygen species (ROS) in cell biology and physiology. Nat. Rev. Mol. Cell. Biol. 23, 499–515. 10.1038/s41580-022-00456-z 35190722

[B147] Sim S. Hibberd M. L. (2016). *Caenorhabditis elegans* susceptibility to gut *Enterococcus faecalis* infection is associated with fat metabolism and epithelial junction integrity. BMC Microbiol. 16, 6. 10.1186/s12866-016-0624-8 26769134 PMC4714453

[B148] Simonis N. Rual J.-F. Carvunis A.-R. Tasan M. Lemmens I. Hirozane-Kishikawa T. (2009). Empirically controlled mapping of the *Caenorhabditis elegans* protein-protein interactome network. Nat. Methods 6, 47–54. 10.1038/nmeth.1279 19123269 PMC3057923

[B149] Sladek F. M. (2011). What are nuclear receptor ligands? Mol. Cell. Endocrinol. 334, 3–13. 10.1016/j.mce.2010.06.018 20615454 PMC3010294

[B150] Sluder A. E. Gilst M. V. Gissendanner C. R. (2002). Diversity and function of orphan nuclear receptors in nematodes. Crit. Rev. Eukaryot. Gene Expr. 12, 24. 10.1615/CritRevEukaryotGeneExpr.v12.i1.40 12433066

[B151] Steinbaugh M. J. Narasimhan S. D. Robida-Stubbs S. Moronetti Mazzeo L. E. Dreyfuss J. M. Hourihan J. M. (2015). Lipid-mediated regulation of SKN-1/Nrf in response to germ cell absence. Elife 4, e07836. 10.7554/eLife.07836 26196144 PMC4541496

[B152] Svensk E. Ståhlman M. Andersson C.-H. Johansson M. Borén J. Pilon M. (2013). PAQR-2 regulates fatty acid desaturation during cold adaptation in *C. elegans* . PLoS Genet. 9, e1003801. 10.1371/journal.pgen.1003801 24068966 PMC3772066

[B153] Svensk E. Devkota R. Ståhlman M. Ranji P. Rauthan M. Magnusson F. (2016). *Caenorhabditis elegans* PAQR-2 and IGLR-2 protect against glucose toxicity by modulating membrane lipid composition. PLoS Genet. 12, e1005982. 10.1371/journal.pgen.1005982 27082444 PMC4833288

[B154] Svensson E. Olsen L. Mörck C. Brackmann C. Enejder A. Faergeman N. J. (2011). The adiponectin receptor homologs in *C. elegans* promote energy utilization and homeostasis. PLoS One 6, e21343. 10.1371/journal.pone.0021343 21712952 PMC3119701

[B155] Tan Q. Wang M. Yu M. Zhang J. Bristow R. G. Hill R. P. (2016). Role of autophagy as a survival mechanism for hypoxic cells in tumors. Neoplasia 18, 347–355. 10.1016/j.neo.2016.04.003 27292024 PMC4909700

[B156] Taubert S. Van Gilst M. R. Hansen M. Yamamoto K. R. (2006). A Mediator subunit, MDT-15, integrates regulation of fatty acid metabolism by NHR-49-dependent and -independent pathways in *C. elegans* . Genes. Dev. 20, 1137–1149. 10.1101/gad.1395406 16651656 PMC1472473

[B157] Taubert S. Ward J. D. Yamamoto K. R. (2011). Nuclear hormone receptors in nematodes: evolution and function. Mol. Cell. Endocrinol. 334, 49–55. 10.1016/j.mce.2010.04.021 20438802 PMC3042524

[B158] Tissenbaum H. A. (2012). Genetics, life span, health span, and the aging process in *Caenorhabditis elegans* . J. Gerontol. A Biol. Sci. Med. Sci. 67, 503–510. 10.1093/gerona/gls088 22499764 PMC3410663

[B159] Tóth M. L. Sigmond T. Borsos E. Barna J. Erdélyi P. Takács-Vellai K. (2008). Longevity pathways converge on autophagy genes to regulate life span in *Caenorhabditis elegans* . Autophagy 4, 330–338. 10.4161/auto.5618 18219227

[B160] Tran T. D. Luallen R. J. (2023). An organismal understanding of *C. elegans* innate immune responses, from pathogen recognition to multigenerational resistance. Semin. Cell. Dev. Biol. S1084-9521 (23), 00060–00065. 10.1016/j.semcdb.2023.03.005 PMC10517082 36966075

[B161] Tullet J. M. A. Hertweck M. An J. H. Baker J. Hwang J. Y. Liu S. (2008). Direct inhibition of the longevity-promoting factor SKN-1 by insulin-like signaling in *C. elegans* . Cell. 132, 1025–1038. 10.1016/j.cell.2008.01.030 18358814 PMC2367249

[B162] Valko A. Perez-Pandolfo S. Sorianello E. Brech A. Wappner P. Melani M. (2021). Adaptation to hypoxia in *Drosophila melanogaster* requires autophagy. Autophagy 18, 909–920. 10.1080/15548627.2021.1991191 34793268 PMC9037493

[B163] Van Gilst M. R. Hadjivassiliou H. Jolly A. Yamamoto K. R. (2005a). Nuclear hormone receptor NHR-49 controls fat consumption and fatty acid composition in *C. elegans* . PLoS Biol. 3, e53. 10.1371/journal.pbio.0030053 15719061 PMC547972

[B164] Van Gilst M. R. Hadjivassiliou H. Yamamoto K. R. (2005b). From the cover: a *Caenorhabditis elegans* nutrient response system partially dependent on nuclear receptor NHR-49. Proc. Natl. Acad. Sci. 102, 13496–13501. 10.1073/pnas.0506234102 16157872 PMC1201344

[B165] Visvikis O. Ihuegbu N. Labed S. A. Luhachack L. G. Alves A.-M. F. Wollenberg A. C. (2014). Innate host defense requires TFEB-mediated transcription of cytoprotective and antimicrobial genes. Immunity 40, 896–909. 10.1016/j.immuni.2014.05.002 24882217 PMC4104614

[B166] Vozdek R. Long Y. Ma D. K. (2018). The receptor tyrosine kinase HIR-1 coordinates HIF-independent responses to hypoxia and extracellular matrix injury. Sci. Signal 11, eaat0138. 10.1126/scisignal.aat0138 30279166

[B167] Walker G. Houthoofd K. Vanfleteren J. R. Gems D. (2005). Dietary restriction in *C. elegans*: from rate-of-living effects to nutrient sensing pathways. Mech. Ageing Dev. 126, 929–937. 10.1016/j.mad.2005.03.014 15896824

[B168] Wallace S. W. Lizzappi M. C. Magemizoğlu E. Hur H. Liang Y. Shaham S. (2021). Nuclear hormone receptors promote gut and glia detoxifying enzyme induction and protect *C. elegans* from the mold P. brevicompactum. Cell. Rep. 37, 110166. 10.1016/j.celrep.2021.110166 34965433 PMC8733895

[B169] Wang M. C. O’Rourke E. J. Ruvkun G. (2008). Fat metabolism links germline stem cells and longevity in *C. elegans* . Science 322, 957–960. 10.1126/science.1162011 18988854 PMC2760269

[B170] Wang X. Jiang Q. Song Y. He Z. Zhang H. Song M. (2022). Ageing induces tissue-specific transcriptomic changes in *Caenorhabditis elegans* . EMBO J. 41, e109633. 10.15252/embj.2021109633 35253240 PMC9016346

[B171] Wani K. A. Goswamy D. Taubert S. Ratnappan R. Ghazi A. Irazoqui J. E. (2021). NHR-49/PPAR-α and HLH-30/TFEB cooperate for *C. elegans* host defense via a flavin-containing monooxygenase. Elife 10, e62775. 10.7554/eLife.62775 33978570 PMC8139828

[B172] Ward J. D. Mullaney B. Schiller B. J. He L. D. Petnic S. E. Couillault C. (2014). Defects in the *C. elegans* acyl-CoA synthase, acs-3, and nuclear hormone receptor, nhr-25, cause sensitivity to distinct, but overlapping stresses. PLoS One 9, e92552. 10.1371/journal.pone.0092552 24651852 PMC3961378

[B173] Watterson A. Arneaud S. L. B. Wajahat N. Wall J. M. Tatge L. Beheshti S. T. (2022a). Loss of heat shock factor initiates intracellular lipid surveillance by actin destabilization. Cell. Rep. 41, 111493. 10.1016/j.celrep.2022.111493 36261024 PMC9642076

[B174] Watterson A. Tatge L. Wajahat N. Arneaud S. L. B. Solano Fonseca R. Beheshti S. T. (2022b). Intracellular lipid surveillance by small G protein geranylgeranylation. Nature 605, 736–740. 10.1038/s41586-022-04729-7 35585236 PMC9885440

[B175] Watts J. L. Browse J. (2002). Genetic dissection of polyunsaturated fatty acid synthesis in *Caenorhabditis elegans* . Proc. Natl. Acad. Sci. U. S. A. 99, 5854–5859. 10.1073/pnas.092064799 11972048 PMC122866

[B176] Watts J. L. Ristow M. (2017). Lipid and carbohydrate metabolism in *Caenorhabditis elegans* . Genetics 207, 413–446. 10.1534/genetics.117.300106 28978773 PMC5629314

[B177] Weikum E. R. Liu X. Ortlund E. A. (2018). The nuclear receptor superfamily: a structural perspective. Protein Sci. 27, 1876–1892. 10.1002/pro.3496 30109749 PMC6201731

[B178] Weir H. J. Yao P. Huynh F. K. Escoubas C. C. Goncalves R. L. Burkewitz K. (2017). Dietary restriction and AMPK increase lifespan via mitochondrial network and peroxisome remodeling. Cell. Metab. 26, 884–896. 10.1016/j.cmet.2017.09.024 29107506 PMC5718936

[B179] Wilson C. González-Billault C. (2015). Regulation of cytoskeletal dynamics by redox signaling and oxidative stress: implications for neuronal development and trafficking. Front. Cell. Neurosci. 9, 381. 10.3389/fncel.2015.00381 26483635 PMC4588006

[B180] Wilson C. Muñoz-Palma E. González-Billault C. (2018). From birth to death: a role for reactive oxygen species in neuronal development. Semin. Cell. Dev. Biol. 80, 43–49. 10.1016/j.semcdb.2017.09.012 28899716

[B181] Zeng L. Li X. Preusch C. B. He G. J. Xu N. Cheung T. H. (2021). Nuclear receptors NHR-49 and NHR-79 promote peroxisome proliferation to compensate for aldehyde dehydrogenase deficiency in *C. elegans* . PLoS Genet. 17, e1009635. 10.1371/journal.pgen.1009635 34237064 PMC8291716

[B182] Zhang Y.-P. Zhang W.-H. Zhang P. Li Q. Sun Y. Wang J.-W. (2022). Intestine-specific removal of DAF-2 nearly doubles lifespan in *Caenorhabditis elegans* with little fitness cost. Nat. Commun. 13, 6339. 10.1038/s41467-022-33850-4 36284093 PMC9596710

[B183] Zhou L. Tong H. Tang H. Pang S. (2021). Fatty acid desaturation is essential for *C. elegans* longevity at high temperature. Mech. Ageing Dev. 200, 111586. 10.1016/j.mad.2021.111586 34655615

[B184] Zuryn S. Kuang J. Tuck A. Ebert P. R. (2010). Mitochondrial dysfunction in *Caenorhabditis elegans* causes metabolic restructuring, but this is not linked to longevity. Mech. Ageing Dev. 131, 554–561. 10.1016/j.mad.2010.07.004 20688098

